# Urinary Metabolomic Changes Accompanying Albuminuria Remission following Gastric Bypass Surgery for Type 2 Diabetic Kidney Disease

**DOI:** 10.3390/metabo12020139

**Published:** 2022-02-02

**Authors:** William P. Martin, Daniel Malmodin, Anders Pedersen, Martina Wallace, Lars Fändriks, Cristina M. Aboud, Tarissa B. Zanata Petry, Lívia P. Cunha da Silveira, Ana C. Calmon da Costa Silva, Ricardo V. Cohen, Carel W. le Roux, Neil G. Docherty

**Affiliations:** 1 Diabetes Complications Research Centre, School of Medicine, Conway Institute of Biomolecular and Biomedical Research, University College Dublin, Belfield, D04 V1W8 Dublin, Ireland; william.martin@ucd.ie (W.P.M.); carel.leroux@ucd.ie (C.W.l.R.); 2 Swedish NMR Centre, University of Gothenburg, 40530 Gothenburg, Sweden; daniel.malmodin@nmr.gu.se (D.M.); anders.pedersen@nmr.gu.se (A.P.); 3 Institute of Food and Health, School of Agriculture and Food Science, University College Dublin, Belfield, D04 V1W8 Dublin, Ireland; martina.wallace@ucd.ie; 4 Institute of Clinical Sciences, Sahlgrenska Academy, University of Gothenburg, 40530 Gothenburg, Sweden; lars.fandriks@gastro.gu.se; 5 The Centre for Obesity and Diabetes, Oswaldo Cruz German Hospital, São Paulo 01333-010, Brazil; mamedio.cristina@gmail.com (C.M.A.); tpetry@haoc.com.br (T.B.Z.P.); lpcunha@haoc.com.br (L.P.C.d.S.); calmon81@hotmail.com (A.C.C.d.C.S.); ricardo.cohen@haoc.com.br (R.V.C.); 6 Diabetes Research Group, Ulster University, Coleraine BT52 1SA, UK

**Keywords:** albuminuria, bariatric surgery, BCAA, chronic kidney disease, diabetic kidney disease, gastric bypass, metabolomics, NMR spectroscopy, obesity, type 2 diabetes

## Abstract

In the Microvascular Outcomes after Metabolic Surgery randomised clinical trial (MOMS RCT, NCT01821508), combined metabolic surgery (gastric bypass) plus medical therapy (CSM) was superior to medical therapy alone (MTA) as a means of achieving albuminuria remission at 2-year follow-up in patients with obesity and early diabetic kidney disease (DKD). In the present study, we assessed the urinary ^1^H-NMR metabolome in a subgroup of patients from both arms of the MOMS RCT at baseline and 6-month follow-up. Whilst CSM and MTA both reduced the urinary excretion of sugars, CSM generated a distinctive urinary metabolomic profile characterised by increases in host–microbial co-metabolites (N-phenylacetylglycine, trimethylamine N-oxide, and 4-aminobutyrate (GABA)) and amino acids (arginine and glutamine). Furthermore, reductions in aromatic amino acids (phenylalanine and tyrosine), as well as branched-chain amino acids (BCAAs) and related catabolites (valine, leucine, 3-hydroxyisobutyrate, 3-hydroxyisovalerate, and 3-methyl-2-oxovalerate), were observed following CSM but not MTA. Improvements in BMI did not correlate with improvements in metabolic and renal indices following CSM. Conversely, urinary metabolites changed by CSM at 6 months were moderately to strongly correlated with improvements in blood pressure, glycaemia, triglycerides, and albuminuria up to 24 months following treatment initiation, highlighting the potential involvement of these shifts in the urinary metabolomic profile in the metabolic and renoprotective effects of CSM.

## 1. Introduction

Diabetic kidney disease (DKD) affects up to 40% of people with longstanding type 2 diabetes mellitus and is the leading cause of end-stage renal disease [1,2]. Whilst renin–angiotensin–aldosterone system blockade has been the backbone of DKD treatment over the last two decades [3], a significant residual risk of progressive renal disease and accelerated cardiovascular disease has remained in people with DKD despite intensive treatment [4]. Recently, two new drug classes have been added to the pharmacological armamentarium, namely sodium–glucose co-transporter-2 inhibitors [5,6] and GLP-1 receptor analogues [7]. The evidence base for the endothelin-A receptor antagonist atrasentan [8] and the nonsteroidal, selective mineralocorticoid receptor antagonist finerenone [9] is following closely behind. Together, these medications promise to improve outcomes.

Epidemiological data from healthcare registries, longitudinal cohorts, and twin analyses establish obesity as an independent risk factor for the onset and progression of chronic kidney disease (CKD), including DKD [10,11,12,13]. Given the high prevalence of obesity in people with CKD, with reported rates ranging from 35–44% [14,15], dedicated obesity treatments constitute a promising means of improving cardiovascular and renal outcomes in this setting. As the most efficacious weight loss intervention, a growing body of research has focused on the renoprotective and cardioprotective effects of bariatric surgery in patients with DKD [16,17,18].

We recently reported the 2-year outcomes of the Microvascular Outcomes after Metabolic Surgery (MOMS) randomised clinical trial (RCT), comparing the impact of combined metabolic surgery plus medical therapy (CSM) versus intensive medical therapy alone (MTA) on urinary albumin excretion in patients with DKD and a body-mass index (BMI) in the 30–35 kg/m^2^ range (NCT01821508). Roux-en-Y gastric bypass surgery (RYGB) was the metabolic surgery employed [19]. Remission of microalbuminuria occurred in 55% (95% CI, 39–70%) of patients after MTA and in 82% (95% CI, 72–93%) of patients after CSM at 2-year follow-up [20].

Measurement of urinary albumin excretion in and of itself provides an incomplete picture of renal parenchymal responses to RYGB. To this end, we have shown in preclinical rat models of DKD that the impact of RYGB on albuminuria is reflective of structural improvements in the glomerulus and the emergence of a reparative pattern of global changes in the renal cortical transcriptome [21,22,23,24]. Whilst histological and molecular assessment of renal tissue post-RYGB in humans is not feasible, assessing changes in urinary metabolite excretion is. Such analysis has the potential to be of hypothesis-generating value with respect to mechanisms and mediators of reductions in renal injury following the procedure, and could also yield patterns of metabolite change of prognostic utility.

The present study thus undertook to profile trajectories of change in urinary metabolite excretion occurring between baseline and 6-month follow-up in a subgroup of patients from both arms of the MOMS RCT. We aimed to characterise the discrete early changes in the urinary metabolome associated with CSM, with the intention of identifying early metabolomic shifts predictive of, and potentially functionally related to, the more favourable metabolic and renal prognosis associated with this intervention at 2-year follow-up.

## 2. Results

### 2.1. Baseline Characteristics of MOMS RCT Patients Included in Sub-Study

Table 1 documents baseline clinical and biochemical parameters for participants in the sub-study. Age and gender were comparable across study arms, although the prevalence of Caucasian ethnicity was higher in the CSM arm compared with the MTA arm (92% vs. 64%, *p* = 0.05). No differences were observed between the study arms at baseline in terms of anthropometry, blood pressure, blood lipids, glycated haemoglobin (HbA_1c_), serum creatinine, or urinary albumin-to-creatinine ratio (uACR). Central tendencies for metabolic and renal parameters characterised the study population as being patients with class I obesity (BMI of 30–35 kg/m^2^), inadequately controlled type 2 diabetes mellitus (HbA_1c_ ≥ 48 mmol/mol or 6.5%), microalbuminuria (uACR 30–300 mg/g), and preserved renal function (mean serum creatinine ~0.8 mg/dL).

### 2.2. Improvements in Metabolic and Renal Parameters Amongst Patients Included in Sub-Study

Relative to baseline, after 6 and 24 months of follow-up, patients included in both arms of the sub-study showed significant reductions in uACR, occurring in the context of improvements in glycaemic control and dyslipidaemia, and, in the case of the CSM group, a median 26% reduction in BMI (Table 2). Amongst patients included in the sub-study, improvements in lipid indices and uACR were similar between the CSM and MTA arms at months 6 and 24. Reductions in BMI, systolic blood pressure, and HbA_1c_ were greater at 6-month follow-up in the CSM arm compared with the MTA arm, with the median 26% reduction in BMI observed in the CSM arm sustained out to month 24 (*p* < 0.001 for comparison with the median 6% reduction in BMI observed in the MTA arm at the same timepoint).

### 2.3. Overview of the Urinary ^1^H-NMR Metabolome and Quality Control Metrics

**Figure 1:**
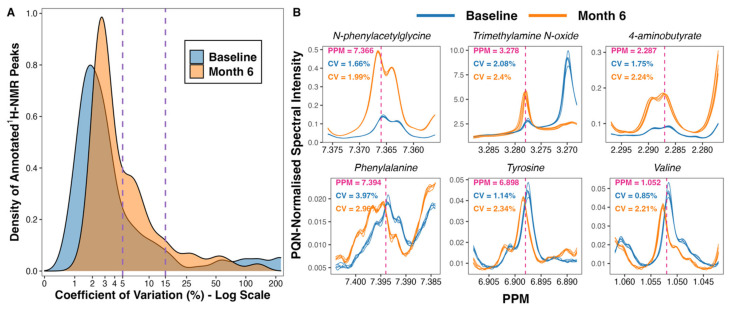
Variance in peak intensity amongst baseline (*n* = 5) and month 6 (*n* = 5) pooled samples included as internal quality controls. (**A**) Density plot outlining the distribution of coefficient of variation values for PQN-normalised peak intensity of annotated ^1^H-NMR peaks (*n* = 208). The percentage coefficient of variation was calculated as follows for each peak: (standard deviation of peak intensity/mean peak intensity) × 100. Calculations were performed separately for baseline and for month 6 pooled samples. The x-axis was log-transformed using the ‘log1p’ transformation from the R package scales [25]. The vertical dashed lines demarcate coefficient of variation values of 5% and 15%. (**B**) Plots of PQN-normalised spectra of pooled samples for certain peaks found to be changed by CSM by multivariate analysis. Plots were generated using the function ‘ROIplot’ from the R package speaq [26]. The vertical dashed lines outline the PPM shift for each peak on the x-axis. Coefficient of variation values for baseline (blue) and for month 6 (orange) pooled samples are superimposed on the plots. ^1^H-NMR, proton nuclear magnetic resonance spectroscopy; CV, coefficient of variation (%); PPM, parts per million chemical shift relative to TSP-d4; PQN, probabilistic quotient normalization.

A total of 475 peaks were detected in the ^1^H-NMR spectra. From these, 208 were annotated, yielding a urinary metabolome consisting of 70 unique metabolites. The coefficient of variation in PQN-normalised peak intensity amongst baseline (*n* = 5) and month 6 (*n* = 5) pooled samples was less than 5% for many, and less than 15% for the majority, of annotated ^1^H-NMR peaks (Figure 1A). Illustrative plots of PQN-normalised spectra of the pooled samples for certain peaks found to be changed by CSM by multivariate analysis demonstrate that the variance of baseline and month 6 pooled samples was low (Figure 1B). Thus, the acquisition of ^1^H-NMR data was technically satisfactory, with acceptable levels of variation observed between pooled samples included as internal quality controls.

### 2.4. Principal Component Analysis of Urinary ^1^H-NMR Peaks

**Figure 2:**
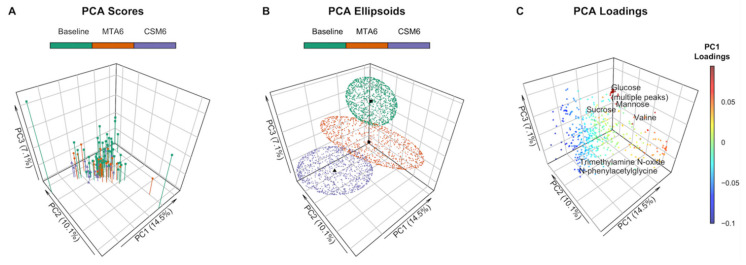
Principal component analysis (PCA) of PQN-normalised urinary ^1^H-NMR peaks for all available samples (*n* = 97). Number of samples by group: baseline, *n* = 54; CSM6, *n* = 19; MTA6, *n* = 24. The first three principal components are presented, with percentages of the total variance explained by each of these principal components outlined in the axis titles. PCA scores and ellipsoids are stratified by study groups. Baseline samples from the CSM and MTA arms were considered together. (**A**) PCA scores. PCA scores are indicated by the points, vertical lines outline the x-y location of points; (**B**) PCA ellipsoids. PCA ellipsoid points were calculated using the function ‘makeEllipsoid’ from the R package ChemoSpec [27], and are presented to more clearly delineate discrete clustering between groups of samples. Centroids (mean PCA scores) are highlighted for the three groups of samples by black points; (**C**) PCA loadings with selected peaks labelled. Points are coloured according to loading values along principal component 1. CSM6, month 6 samples from CSM group; MTA6, month 6 samples from MTA group; PC, principal component; PCA, principal component analysis; PQN, probabilistic quotient normalization.

Clustering along the first three principal components identified discrete between-group changes in the urinary metabolome (Figure 2A,B). Three outlying samples (two baseline, one MTA6) were identified on the scores plot (Figure 2A). These samples were included in multivariate analyses as excluding them did not appreciably alter the performance of the partial least squares (PLS) models nor the peaks identified as being important to the models. The most variability was observed in baseline samples, which, from the loadings, was identified as being primarily due to excursions in sugars such as glucose, mannose, and sucrose prior to intensification of treatment (Figure 2C). The branched-chain amino acid (BCAA) valine also contributed to dispersion in baseline samples. MTA6 samples clustered more closely with baseline samples than CSM6 samples. Separation of CSM6 samples from the other samples was identified as being attributable to an increased abundance of metabolites such as N-phenylacetylglycine and trimethylamine N-oxide on the loadings plot.

### 2.5. Fold Change Differences in Urinary ^1^H-NMR Peak Intensity by Univariate Testing

**Figure 3:**
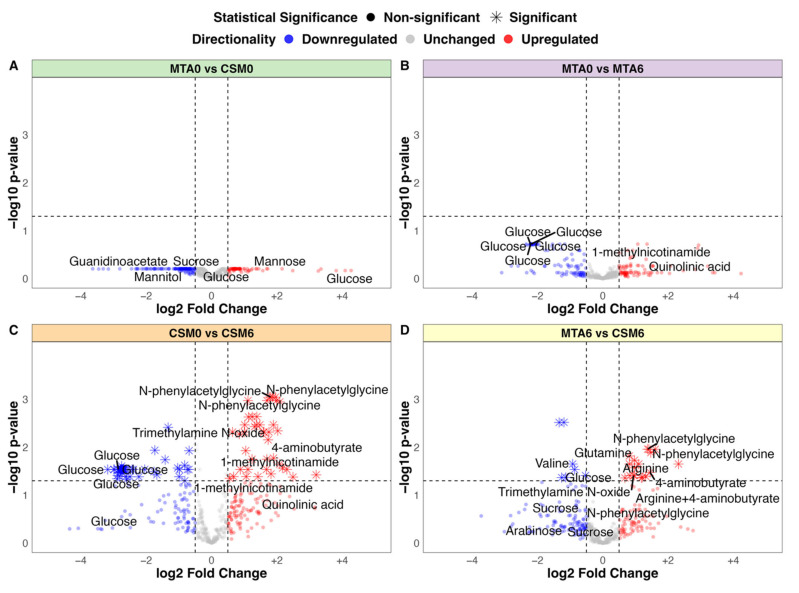
Volcano plots of between-group fold change differences in urinary ^1^H-NMR peaks (all available samples). (**A**) MTA0 (*n* = 28) vs. CSM0 (*n* = 26); (**B**) MTA0 (*n* = 28) vs. MTA6 (*n* = 24); (**C**) CSM0 (*n* = 26) vs. CSM6 (*n* = 19); (**D**) MTA6 (*n* = 24) vs. CSM6 (*n* = 19). Log2 fold change values and adjusted *p*-values were derived from unpaired *t*-tests of between-group differences in peak intensity. Log2 fold change is presented on the x-axis with –log10 transformation of the multiplicity-corrected (Benjamini–Hochberg) *p*-value for between-group differences on the y-axis. Log2 fold change values and adjusted *p*-values derived from univariate testing of the CSM0 vs. CSM6 and MTA6 vs. CSM6 comparisons are also summarised in Table 3. A selection of the most strongly changed peaks for each comparison are labelled with metabolite annotations. Dots are coloured by directionality of change: blue = downregulated (log2 fold change < −0.5); grey = unchanged (log2 fold change > −0.5 and < 0.5); red = upregulated (log2 fold change > 0.5). Horizontal and vertical lines demarcate thresholds used to define metabolites with strong between-group changes: log2 fold change values of −0.5 and 0.5, as well as a −log10 *p*-value of 1.3 (corresponding to an absolute *p*-value of 0.05). CSM0, baseline samples from CSM group; CSM6, month 6 samples from CSM group; MTA0, baseline samples from MTA group; MTA6, month 6 samples from MTA group.

No significant differences in urinary metabolite abundance were observed between baseline samples from the MTA and CSM groups by univariate testing (Figure 3A). The strongest change in MTA samples from baseline to month 6 was a non-statistically significant reduction in urinary glucose excretion (Figure 3B). The magnitude of the reduction in urinary glucose excretion was greater from baseline to month 6 in CSM samples, and reached statistical significance (Figure 3C). Furthermore, exclusive increases in the urinary excretion of a range of metabolites occurred following CSM. Most notable among these were several peaks containing N-phenylacetylglycine, trimethylamine N-oxide, 4-aminobutyrate (GABA), and 1-methylnicotinamide. Consequently, the main differences between month 6 samples from the MTA and CSM arms related to increased urinary metabolite excretion following RYGB, including the aforementioned metabolites as well as the amino acids arginine and glutamine (Figure 3D). Compared with the MTA arm, urinary concentrations of sugars such as sucrose and arabinose, as well as the BCAA valine, were also lower in the CSM arm at month 6.

### 2.6. Baseline Urinary Metabolomic Differences between Study Arms: MTA0 vs. CSM0

The PLS classification model for baseline between-group differences performed better than expected, although it was still quite weak with an AUC of 0.7 (Supplementary Figure S1A). The PLS model misclassified 17 of 54 samples, resulting in an overall accuracy of 69% (Figure S1B), with the all-relevant ‘max’ MUVR model identifying over 200 ^1^H-NMR peaks as potentially useful in classifying CSM and MTA samples at baseline (Figure S1C). The actual PLS model misclassified fewer samples than permuted models with a randomly sampled Y response vector (*p* = 0.01; Figure S1D). Many of the peaks important to model performance were uraemic toxins (phenol, guanidinoacetate) or sugars (fucose, mannitol, sucrose), although the directionality of change of these two groups of metabolites was not consistent between the two study arms (Figure S1E). Furthermore, none of the metabolites identified by this model were significantly different between both arms at baseline by univariate testing (Figure 3A). These subtle alterations in urinary metabolomic profiles were thus not indicative of a systematic difference in disease severity at baseline between the CSM and MTA arms.

### 2.7. Urinary Metabolites Attributable to Differences in Ethnicity in the MTA Arm: Caucasian vs. Other Ethnicities in MTA0 Samples

As there were differences in ethnicity in the MTA arm at baseline (Table 1; 64% were Caucasian and 36% were of other ethnicity, including South American, Asian, and African), a PLS classification model of Caucasian (*n* = 18) versus other ethnicities (*n* = 10) was constructed to examine for ethnicity-related differences in the urinary metabolome in baseline MTA samples. The PLS model had a high misclassification rate (18 of 28 samples misclassified; overall classification accuracy of 36%) and low sensitivity (50%) and specificity (10%; Figure S2A), despite optimization of validation performance (Figure S2B). The actual PLS model did not perform better than permuted models with a randomly sampled Y response vector (*p* = 0.97; Figure S2C). Overall, the PLS model provided no evidence to suggest a systematic difference in urinary metabolomic profiles between individuals of Caucasian and other ethnicities.

### 2.8. Changes in the Urinary Metabolome Following MTA: MTA0 vs. MTA6

The PLS model using all available samples between baseline and month 6 in the MTA arm was quite weak, with an AUC of 0.68 (Figure S3A). The model misclassified 19 of 52 samples, resulting in an overall accuracy of 63% (Figure S3B), despite optimization of the validation performance of the ‘max’ model by minimising the number of misclassifications (Figure S3C). The actual PLS model did not have a lower misclassification rate than randomly permuted models (*p* = 0.06; Figure S3D). The two annotated peaks that were most important to model performance are both involved in nicotinamide metabolism, namely 1-methylnicotinamide and trigonelline (N-methylnicotinate) (Figure S3E). Urinary excretion of 1-methylnicotinamide increased following MTA, while that of trigonelline decreased. Otherwise, the main differences between MTA samples from baseline to month 6 were related to a reduction in urinary excretion of glucose and related metabolites. However, none of the metabolites identified by this model were significantly different between MTA0 and MTA6 samples by univariate testing (Figure 3B).

The PLS model, which used only paired samples between baseline and month 6 in the MTA arm, was weak, with an AUC of 0.56 (Figure S4A) and an overall classification accuracy of 46% (Figure S4B), despite training and tuning of model parameters to optimise validation performance (Figure S4C). The number of misclassifications by this model was not lower than that achieved by randomly permuted models (*p* = 0.59; Figure S4D). When the abundance of all metabolites in month 6 samples from the MTA arm was considered together, for example by principal component analysis, it was possible to distinguish them from baseline samples (Figure 2A). However, the identification of individual metabolites which allowed for accurate prediction of baseline and month 6 status in the MTA arm proved difficult, indicating that changes in individual metabolites in the MTA arm were generally weak. Accordingly, variable importance in projection (VIP) values for the top 20 metabolites contributing to the MTA0 vs. MTA6 paired samples PLS model were higher (indicating lower variable importance) than for the top 20 metabolites contributing to other PLS models (Figure S4E), particularly those incorporating month 6 samples from the CSM arm.

### 2.9. Changes in the Urinary Metabolome Following CSM: CSM0 vs. CSM6

**Figure 4:**
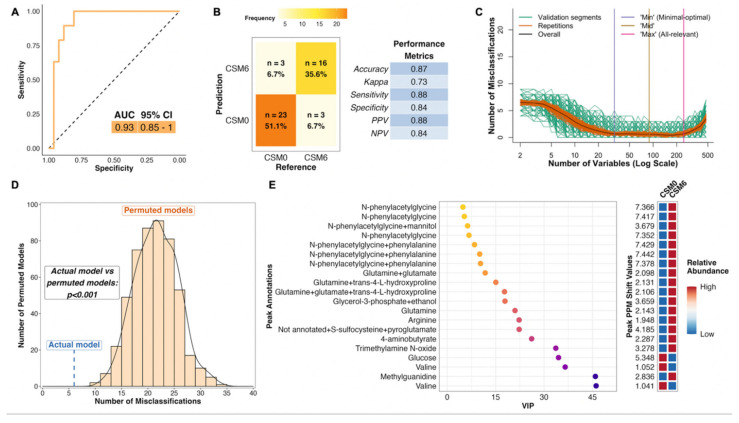
Multivariate analysis of changes in the urinary metabolome from baseline to month 6 in the CSM arm (all available samples). Number of samples inputted to the MUVR PLS model: CSM0, *n* = 26; CSM6, *n* = 19. (**A**) Receiver operating characteristic curve for the MUVR PLS classification model fit to urinary ^1^H-NMR peaks for these two groups of samples. Inset are the AUC and associated 95% CI for the curve. (**B**) Confusion matrix outlining sample classification by the PLS model. The x-axis presents the actual class of the inputted samples. The y-axis presents the sample class predicted by the models. Correctly and incorrectly classified samples are outlined on opposing diagonals of the confusion matrix. Additional performance metrics relating to sample classification at the 50% probability threshold are presented in the table adjacent to the confusion matrix. (**C**) Recursive ranking and backward elimination of variables (right-to-left on the x-axis) in the inner segments of the MUVR PLS model to achieve optimal validation performance, quantified by the number of misclassifications (y-axis). Green lines represent validation curves per inner segment and may fluctuate. Orange and black lines represent inner segment curves averaged per model repetition and overall (100 model repetitions), respectively, and describe the actual validation performance at higher resolution. Vertical lines outline the number of variables selected in MUVR ‘min’, ‘mid’, and ‘max’ models on the x-axis. (**D**) Histogram and density curve outlining the distribution of misclassifications by 500 permuted PLS models, in which the Y response vector indicating sample class was randomly sampled. The number of misclassifications by the actual PLS model with the correct Y response vector is outlined on the x-axis by the blue dashed line. A *p*-value derived from a Student’s *t*-test comparing the number of misclassifications between actual and permuted models is presented adjacent to the histogram and density curve. (**E**) Dot plot of the top 20 most important annotated peaks to performance of the PLS model ranked by VIP values. Multiple peaks were identified for certain metabolites. When multiple metabolites are present in a given peak, metabolites are listed in order of relative abundance in the peak, with the most abundant metabolite listed first. The adjacent heatmap illustrates relative abundance of metabolites based on mean peak intensity across all samples in the two groups evaluated by the PLS model. 95% CI, 95% confidence interval; AUC, area under the receiver operating characteristic curve; CSM0, baseline samples from CSM group; CSM6, month 6 samples from CSM group; MUVR, multivariate methods with unbiased variable selection in R; NMR, nuclear magnetic resonance spectroscopy; NPV, negative predictive value; PLS, partial least squares; PPM, parts per million chemical shift relative to TSP-d4; PPV, positive predictive value; VIP, variable importance in projection.

The PLS model using all available samples between baseline and month 6 in the CSM arm performed much better than the corresponding model for the MTA arm, with an AUC of 0.93 (Figure 4A). The model misclassified 6 of 45 samples, resulting in an overall accuracy of 87%, sensitivity of 88%, and specificity of 84% (Figure 4B). The number of misclassifications was also effectively minimized to approximately zero during optimization of validation performance (Figure 4C). The performance of the actual CSM0 vs. CSM6 PLS model was significantly better than randomly permuted models (*p* < 0.001; Figure 4D).

Thus, distinctive metabolomic changes occurred following RYGB, which allowed for accurate classification of baseline and month 6 samples in the CSM arm. The top seven most important annotated peaks for model performance all contained the host–microbial co-metabolite N-phenylacetylglycine, the urinary excretion of which was increased following CSM (Figure 4E). Increased urinary excretion of other host–microbial co-metabolites, including trimethylamine N-oxide and 4-aminobutyrate (GABA), was observed following CSM. Urinary excretion of the amino acids glutamine and arginine also increased following CSM, while that of glucose and the BCAA valine decreased. Log2 fold change values and adjusted *p*-values derived from univariate testing of between-group differences in urinary ^1^H-NMR peak intensity for peaks identified as differentially abundant between CSM0 and CSM6 samples are summarized in Table 3 below. VIP metrics from PLS models of the relevant comparisons are also presented in the table.

The PLS model which used only paired samples between baseline and month 6 in the CSM arm performed similarly well to the model that used all available samples from both timepoints, with an AUC of 0.94 (Figure S5A) and an overall classification accuracy of 82% (Figure S5B). Similar to the CSM0 vs. CSM6 PLS model using all available samples, the validation performance of this paired samples model was optimized to achieve almost no misclassifications (Figure S5C). The number of misclassifications by the actual paired samples PLS model was significantly lower than randomly permuted models (*p* = 0.006; Figure S5D).

Both CSM0 vs. CSM6 PLS models, using all available samples and just paired samples, identified very similar metabolites as being important to the distinction of baseline and month 6 samples in the CSM arm. Several N-phenylacetylglycine peaks were again identified as the most important annotated peaks to performance of the paired samples PLS model (Figure S5E). The urinary excretion of the majority of metabolites identified as important to model performance increased following CSM, with the exception of phenylalanine and valine, whose urinary excretion decreased following CSM.

### 2.10. Differences in the Urinary Metabolome between CSM and MTA Arms after 6 Months of Treatment: MTA6 vs. CSM6

**Figure 5:**
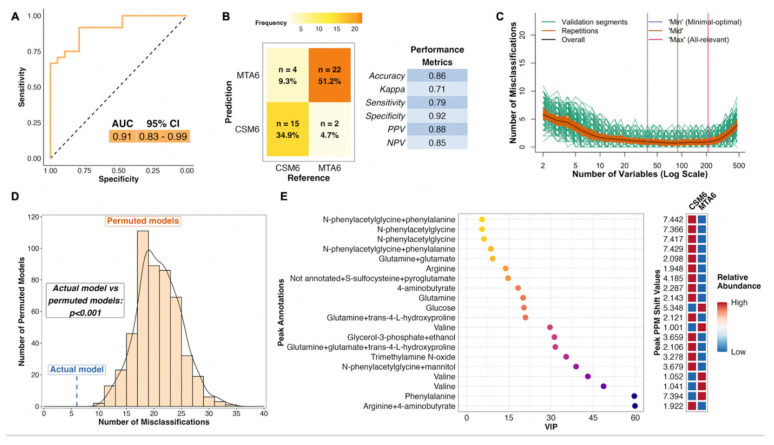
Multivariate analysis of between-group differences in the urinary metabolome at month 6 (all available samples). Number of samples inputted to the MUVR PLS model: CSM6, *n* = 19; MTA6, *n* = 24. (**A**) Receiver operating characteristic curve for the MUVR PLS classification model fit to urinary ^1^H-NMR peaks for these two groups of samples. Inset are the AUC and associated 95% CI for the curve. (**B**) Confusion matrix outlining sample classification by the PLS model. The x-axis presents the actual class of the inputted samples. The y-axis presents the sample class predicted by the models. Correctly and incorrectly classified samples are outlined on opposing diagonals of the confusion matrix. Additional performance metrics relating to sample classification at the 50% probability threshold are presented in the table adjacent to the confusion matrix. (**C**) Recursive ranking and backward elimination of variables (right-to-left on the x-axis) in the inner segments of the MUVR PLS model to achieve optimal validation performance, quantified by the number of misclassifications (y-axis). Green lines represent validation curves per inner segment and may fluctuate. Orange and black lines represent inner segment curves averaged per model repetition and overall (100 model repetitions), respectively, and describe the actual validation performance at higher resolution. Vertical lines outline the number of variables selected in MUVR ‘min’, ‘mid’, and ‘max’ models on the x-axis. (**D**) Histogram and density curve outlining the distribution of misclassifications by 500 permuted PLS models, in which the Y response vector indicating sample class was randomly sampled. The number of misclassifications by the actual PLS model with the correct Y response vector is outlined on the x-axis by the blue dashed line. A *p*-value derived from a Student’s *t*-test comparing the number of misclassifications between actual and permuted models is presented adjacent to the histogram and density curve. (**E**) Dot plot of the top 20 most important annotated peaks to performance of the PLS model ranked by VIP values. Multiple peaks were identified for certain metabolites. When multiple metabolites are present in a given peak, metabolites are listed in order of relative abundance in the peak, with the most abundant metabolite listed first. The adjacent heatmap illustrates relative abundance of metabolites based on mean peak intensity across all samples in the two groups evaluated by the PLS model. 95% CI, 95% confidence interval; AUC, area under the receiver operating characteristic curve; CSM6, month 6 samples from CSM group; MTA6, month 6 samples from MTA group; MUVR, multivariate methods with unbiased variable selection in R; NMR, nuclear magnetic resonance spectroscopy; NPV, negative predictive value; PLS, partial least squares; PPM, parts per million chemical shift relative to TSP-d4; PPV, positive predictive value; VIP, variable importance in projection.

The PLS model for month 6 between-group differences performed well, with an AUC of 0.91 (Figure 5A). The model misclassified 6 of 43 samples, resulting in an overall classification accuracy of 86%, a sensitivity of 79%, and a specificity of 92% (Figure 5B). Similar to both of the CSM0 vs. CSM6 PLS models, the validation performance of this model was optimized to achieve almost no misclassifications (Figure 5C). The number of misclassifications by the actual MTA6 vs. CSM6 PLS model was significantly lower than that achieved by permuted models with a randomly sampled Y response vector (*p* < 0.001; Figure 5D).

The metabolites identified as important to the performance of the MTA6 vs. CSM6 model were mainly those whose urinary excretion was strongly changed following CSM and were therefore identified as influential metabolites in terms of classifying baseline and month 6 samples in the CSM arm (Figure 5E). Several peaks containing N-phenylacetylglycine were again identified as the most important peaks for model classification. Urinary concentrations of arginine, glutamine, N-phenylacetylglycine, 4-aminobutyrate, and trimethylamine N-oxide were higher in the CSM arm compared with the MTA arm at month 6. Conversely, urinary excretion of glucose, valine, and phenylalanine was lower in the CSM arm compared with the MTA arm at month 6.

Table 3 presents a summary of log2 fold change values and adjusted *p*-values derived from univariate testing of between-group differences in urinary ^1^H-NMR peak intensity, as well as VIP metrics from PLS classification models of the relevant comparisons, for peaks changed by CSM from baseline to 6-month follow-up. The top 20 annotated peaks, according to VIP rank, identified by PLS models for the CSM0 vs. CSM6 all available samples, CSM0 vs. CSM6 paired samples, and the MTA6 vs. CSM6 all available samples comparisons are presented. There was considerable overlap in the most important peaks across all 3 models, resulting in a total of 25 distinct peaks identified as being changed by CSM. Amongst these 25 peaks, several compounds, such as N-phenylacetylglycine and valine, were represented more than once. For each compound with multiple peaks, a single representative chemical shift was selected to reduce the duplication of information presented in Table 3.

### 2.11. Inter-Peak Correlations and Abundance by Study Arm and Timepoint of Peaks Changed by CSM from Baseline to Month 6

**Figure 6:**
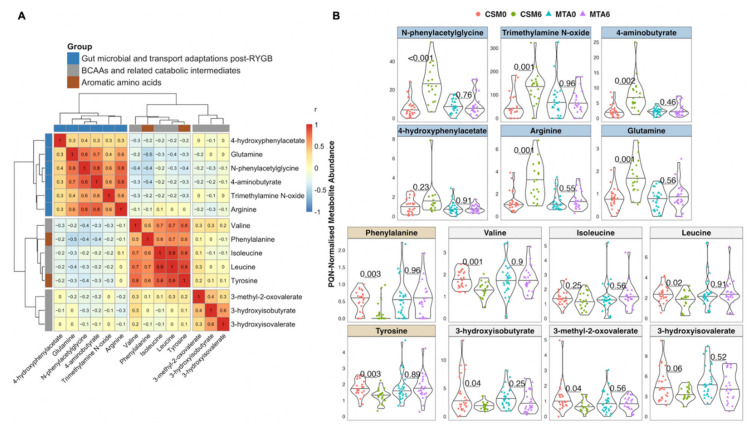
Inter-peak correlations and abundance by study arm and timepoint of peaks changed by CSM from baseline to month 6. (**A**) Heatmap of correlations between PQN-normalised peak intensity values for 14 selected metabolites. Metabolites were selected on the basis of being identified as strongly changed by CSM from baseline to month 6 by multivariate PLS models. Additional metabolites were manually selected on the basis of belonging to similar functional groupings to those metabolites identified by PLS models. Heatmap rows and columns depict individual metabolites, with Pearson r correlation values presented in the heatmap cells. Functional groupings of metabolites are identified by the row and column annotations as follows: blue = metabolites reflective of changes in gut microbial metabolism or gut transport after RYGB; grey = metabolites reflective of BCAA catabolism; brown = aromatic amino acids. Gaps in heatmap rows and columns demarcate clusters of metabolites based on correlation coefficients. (**B**) PQN-normalised abundance by study arm and timepoint of the 14 metabolites outlined in the heatmap in panel (a). Number of samples: CSM0, *n* = 26; CSM6, *n* = 19; MTA0, *n* = 28; MTA6, *n* = 24. The top 2 rows (blue panels) indicate metabolites reflective of changes in gut microbial metabolism or gut transport after RYGB. The bottom 2 rows indicate aromatic amino acids (brown panels) and metabolites reflective of BCAA catabolism (grey panels). *p*-values derived from unpaired *t*-tests of baseline and month 6 differences in peak intensity in the CSM and MTA arms are presented. *p*-values were multiplicity-corrected for the number of comparisons using the Benjamini–Hochberg method. BCAA, branched-chain amino acid; CSM, combined metabolic surgery plus medical therapy; CSM0, baseline samples from CSM group; CSM6, month 6 samples from CSM group; MTA, medical therapy alone; MTA0, baseline samples from MTA group; MTA6, month 6 samples from MTA group; PLS, partial least squares; PQN, probabilistic quotient normalization; RYGB, Roux-en-Y gastric bypass surgery.

Based on the PLS models highlighted above, as well as a manual review of the spectra, 14 metabolites changed by CSM from baseline to month 6 and with related functional groupings were identified. Clustering of inter-peak correlations between these 14 metabolites identified three clusters composed of host–microbial co-metabolites, branched-chain and aromatic amino acids, and BCAA catabolic intermediates (Figure 6A). Urinary concentrations of the various host–microbial co-metabolites were strongly correlated with each other, as were branched-chain and aromatic amino acids. BCAA catabolic intermediates were more weakly correlated with each other. Aromatic amino acids were more strongly correlated with BCAAs than they were with host–microbial co-metabolites, despite the fact that altered host–microbial aromatic amino acid metabolism resulting in the production of metabolites such as N-phenylacetylglycine and 4-hydroxyphenylacetate has been consistently reported following RYGB [28,29,30,31,32,33].

In the CSM arm, the increased urinary excretion from baseline to month 6 of metabolites reflective of changes in gut microbial metabolism and intestinal transport of amino acids following RYGB was marked and strongly statistically significant for N-phenylacetylglycine (*p* < 0.001), trimethylamine N-oxide (*p* = 0.001), 4-aminobutyrate (GABA; *p* = 0.002), arginine (*p* = 0.001), and glutamine (*p* = 0.001) (Figure 6B). The urinary excretion of these metabolites was not altered by MTA.

Similarly, urinary excretion of the aromatic amino acids, phenylalanine (*p* = 0.003) and tyrosine (*p* = 0.003), as well as the BCAAs valine (*p* = 0.001) and leucine (*p* = 0.02), was significantly reduced from baseline to month 6 following CSM but not MTA. Urinary excretion of the BCAA isoleucine also trended to be lower following CSM, but this was not significant (*p* = 0.25). Consistent with the reduced urinary excretion of BCAAs and pointing to enhanced BCAA catabolism, urinary excretion of BCAA catabolic intermediates also decreased following CSM. Urinary concentrations of valine (3-hydroxyisobutyrate; *p* = 0.04), isoleucine (3-methyl-2-oxovalerate; *p* = 0.04), and leucine (3-hydroxyisovalerate; *p* = 0.06) catabolites decreased following CSM but not MTA.

### 2.12. Correlations between Changes in Metabolites Reflective of Host–Microbial Co-Metabolism and BCAA Catabolism with Improvements in Metabolic and Renal Indices Following CSM for DKD

**Figure 7:**
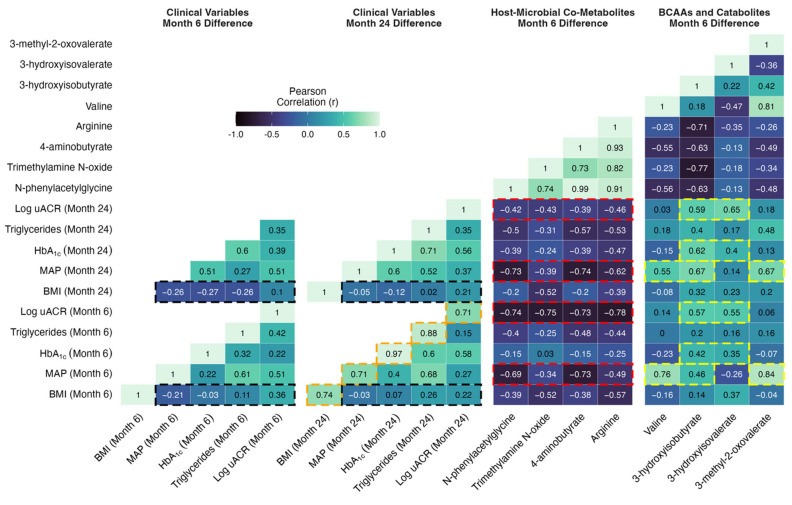
Correlation matrix of changes in clinical parameters at months 6 and 24 with changes in urinary metabolites reflective of host–microbial co-metabolism and BCAA catabolism at month 6. Absolute differences in clinical parameters at both timepoints (months 6 and 24) were correlated with absolute differences in PQN-normalised metabolite abundance at month 6. Correlations were performed for the subset of study participants in the CSM arm with paired urinary ^1^H-NMR data available at baseline and month 6 (*n* = 11). Pearson r correlation values are presented. Selected correlations are highlighted by black, orange, red, and yellow shading around cells of the correlation matrix. *p*-values corresponding to the Pearson correlation r values outlined above are presented in Figure S6. BCAA, branched-chain amino acid; BMI, body-mass index; CSM, combined metabolic surgery plus medical therapy; HbA_1c_, glycated haemoglobin; MAP, mean arterial pressure; PQN, probabilistic quotient normalization; uACR, urinary albumin-to-creatinine ratio.

Improvements in individual metabolic and renal parameters from baseline to month 6 following CSM were strongly associated with the magnitude of improvement observed at month 24. Correlations highlighted by the orange shading in Figure 7 emphasise that r values > 0.7 were observed between month 6 and month 24 changes in individual metabolic and renal indices following CSM, including BMI, MAP, HbA_1c_, triglycerides, and uACR. These correlations were all statistically significant, with *p*-values < 0.05 observed (Figure S6). The relationship between month 6 and month 24 change in HbA_1c_ was particularly strong, with an r value of 0.97 (*p* < 0.001) observed. Thus, the magnitude of early improvements in individual metabolic and renal indices following CSM strongly predicted the magnitude of improvements observed at 2-year follow-up.

Reduction in BMI is one of the most consistent effects of bariatric surgery, and the degree of weight loss is purported to be a principal determinant of improvements in other metabolic parameters. However, as outlined in the cells highlighted in black in Figure 7, absent or weak correlations were observed between changes in BMI at months 6 and 24 with changes in blood pressure, glycaemia, triglycerides, and log uACR at months 6 and 24 in the CSM arm. All *p*-values for these correlations were > 0.25 and non-significant (Figure S6). The strongest such correlations were observed between changes in BMI and in log uACR (month 6, r = 0.36 [*p* = 0.27]; month 24, r = 0.22 [*p* = 0.54]), although these remained quite weak. Thus, in the CSM arm, the amount of weight loss did not correlate strongly with the improvements observed in other metabolic parameters and in renal indices. While improvements in BMI were quite consistent at month 6 following CSM, improvements in other metabolic parameters and in renal indices were more variable, and this variability was not well captured by changes in BMI. We investigated whether changes in urinary metabolite excretion correlated more strongly than changes in BMI with improvements in metabolic and renal indices following CSM.

Moderate to strong inverse correlations were observed between increases in urinary host–microbial co-metabolites (N-phenylacetylglycine, trimethylamine N-oxide, 4-aminobutyrate, and arginine) and reductions in clinical markers of adverse metabolic and renal health in the CSM arm (Figure 7, Figures S6 and S7). As outlined in the cells highlighted in red in Figure 7, these correlations were strongest for improvements in blood pressure and albuminuria at months 6 and 24. Correlations between changes in urinary host–microbial co-metabolites and improvements in BMI, HbA_1c_, and triglycerides were generally weaker, although some of the correlations were moderately strong. N-phenylacetylglycine and 4-aminobutyrate were most strongly correlated with improvements in blood pressure (month 6: r = −0.69 [*p* = 0.03] for N-phenylacetylglycine and r = −0.73 [*p* = 0.02] for 4-aminobutyrate; month 24: r = −0.73 [*p* = 0.02] for N-phenylacetylglycine and r = −0.74 [*p* = 0.02] for 4-aminobutyrate). Correlations with improvements in albuminuria at months 6 and 24 were of similar strength for all four host–microbial co-metabolites. Correlations with log uACR were < −0.7 (all *p*-values < 0.05) for all four metabolites at month 6, and while these weakened by month 24, correlations remained around −0.4 for all four metabolites. Trimethylamine N-oxide was most strongly correlated with improvements in BMI, with an r value of −0.52 observed for both month 6 (*p* = 0.1) and month 24 (*p* = 0.12) changes in BMI. The moderate to strong correlations with improvements in clinical parameters evident for month 6 changes in urinary host–microbial co-metabolites are contrasted against the weak ones observed for month 6 changes in BMI in the correlation scatterplots in Figure S7.

Moderately strong correlations were observed between metabolites reflective of BCAA catabolism (valine plus the BCAA catabolic intermediates 3-hydroxyisobutyrate, 3-hydroxyisovalerate, and 3-methyl-2-oxovalerate) with improvements in blood pressure, HbA_1c_, and albuminuria in the CSM arm (Figure 7, Figure S6 and S8). Correlations between metabolites reflective of BCAA catabolism with BMI and triglycerides were generally weak. As outlined in the cells highlighted in yellow in Figure 7, correlations with improvements in blood pressure at months 6 and 24 were strongest for valine, 3-hydroxyisobutyrate, and 3-methyl-2-oxovalerate (month 6: r = 0.76 [*p* = 0.01] for valine, r = 0.46 for 3-hydroxyisobutyrate [*p* = 0.18], and r = 0.84 [*p* < 0.001] for 3-methyl-2-oxovalerate; month 24: r = 0.55 [*p* = 0.12] for valine, r = 0.67 [*p* = 0.05] for 3-hydroxyisobutyrate, and r = 0.67 [*p* = 0.05] for 3-methyl-2-oxovalerate). Changes in valine and 3-methyl-2-oxovalerate were not significantly correlated with improvements in other clinical parameters. However, as highlighted in the cells outlined in yellow in Figure 7, both 3-hydroxyisobutyrate and 3-hydroxyisovalerate were moderately strongly correlated with improvements in HbA_1c_ and albuminuria at months 6 and 24, with the relationships to log uACR being slightly stronger (month 6: r = 0.57 [*p* = 0.07] for 3-hydroxyisobutyrate and r = 0.55 [*p* = 0.08] for 3-hydroxyisovalerate; month 24: r = 0.59 for 3-hydroxyisobutyrate [*p* = 0.07] and r = 0.65 [*p* = 0.04] for 3-hydroxyisovalerate). The moderately strong correlations with improvements in clinical parameters evident for month 6 changes in urinary metabolites reflective of BCAA catabolism are contrasted against the weak ones observed for month 6 changes in BMI in the correlation scatterplots in Figure S8.

## 3. Discussion

**Figure 8:**
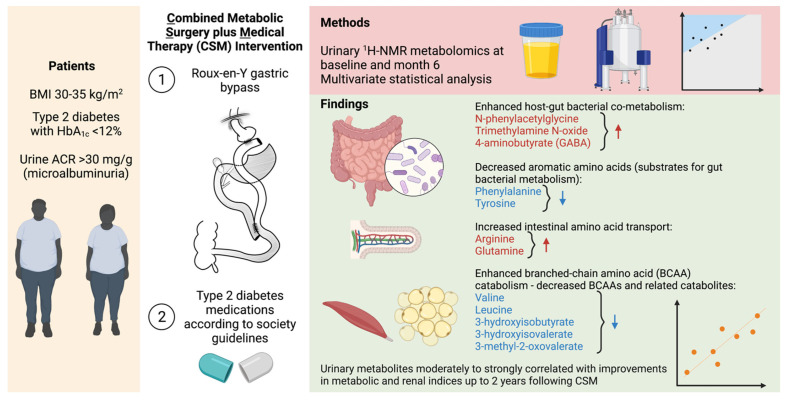
Overview of urinary metabolomic changes occurring at 6 months after CSM. Metabolites that increased from baseline to month 6 are outlined in red while those that decreased are outlined in blue. Created with BioRender.com. ^1^H-NMR, proton nuclear magnetic resonance spectroscopy; ACR, albumin-to-creatinine ratio; BMI, body-mass index; HbA_1c_, glycated haemoglobin.

In this sub-study of the MOMS RCT [20], we interrogated urinary ^1^H-NMR metabolomic changes after treatment with medications alone or with RYGB plus medications in patients with obesity, type 2 diabetes, and microalbuminuria (Figure 8).

Compared with MTA, treatment with CSM resulted in a distinctive urinary metabolomic profile. The urinary metabolomic changes induced by CSM included increased excretion of the host–microbial co-metabolites N-phenylacetylglycine, trimethylamine N-oxide, and 4-aminobutyrate (GABA) [28,29,30,31,32,33], as well as increased excretion of the amino acids arginine and glutamine. CSM also reduced urinary concentrations of aromatic amino acids (phenylalanine and tyrosine) as well as BCAAs (valine and leucine) and related catabolic intermediates (3-hydroxyisobutyrate, 3-hydroxyisovalerate, and 3-methyl-2-oxovalerate). While reductions in both aromatic amino acids and BCAAs may be related to weight loss and improved insulin sensitivity following CSM [34,35,36,37,38,39,40], diminished urinary concentrations of aromatic amino acids may also be directly related to their reduced gut absorption following RYGB, resulting in enhanced metabolism by gut bacteria [30].

The most prominent metabolomic change observed in this study was an increase in the urinary excretion of host–microbial co-metabolites at 6 months after CSM, including N-phenylacetylglycine, trimethylamine N-oxide, 4-aminobutyrate (GABA), and arginine. Importantly, changes in urinary host–microbial co-metabolites following CSM were moderately to strongly correlated with improvements in blood pressure, triglycerides, and uACR up to 2 years following treatment initiation. Increased urinary excretion of host–microbial co-metabolites following RYGB has previously been reported, and ascribed to compositional and functional changes in the gut microbiota, which may account for weight loss-independent metabolic improvements following the procedure [28,29,30,31,32,33,41,42]. Seyfried et al. demonstrated increased urinary excretion of the host–microbial co-metabolites phenylacetylglycine, indoxyl sulphate, and 4-cresyl glucuronide at 2 and 4 weeks after RYGB in an obese Zucker rat model [32]. These changes were not observed following caloric restriction alone in the animal model. We have also previously observed increased urinary excretion of N-phenylacetylglycine and 3-indoxyl sulphate at 4 weeks after both RYGB alone and RYGB combined with fenofibrate, metformin, ramipril, and rosuvastatin in the Zucker Diabetic Sprague Dawley rat model [33]. Increased urinary excretion of N-phenylacetylglycine points to reduced absorption of the aromatic amino acid phenylalanine and consequent enhanced metabolism by gut bacteria following RYGB [32]. Consistent with this, urinary phenylalanine excretion decreased from baseline to month 6 in the CSM arm.

Reduced intestinal motility following RYGB promotes protein putrefaction, with incompletely digested proteins reaching the colon due to surgical bypass of the foregut [29]. As such, increased faecal excretion of GABA has been reported following RYGB in rodent models and is attributed to protein putrefaction and microbial processing of putrescine derived from ornithine [29,32,43]. Increased faecal GABA is implicated in the well-characterised increase in glucagon-like peptide-1 (GLP-1) observed following RYGB, as GABA can stimulate GLP-1 release from enterocytes through ionotropic GABA_A_ and GABA_C_ receptors [44,45]. GLP-1 induced natriuresis is implicated in the antihypertensive and renoprotective effects of RYGB [16,46]. GABA may not only stimulate GLP-1 secretion, but may also exert direct natriuretic effects and lower blood pressure through a local renal GABAergic system [47]. Thus, the potential direct and indirect renoprotective effects of increased urinary GABA excretion observed following CSM in the present study merit further interrogation.

Urinary excretion of arginine and glutamine (a precursor of arginine [48]) also increased following CSM, and urinary concentrations of the two amino acids were moderately strongly correlated with each other (r = 0.6). In a high-fat diet Wistar rat model, RYGB was demonstrated to alter microbial arginine biosynthesis, resulting in lower colonic arginine concentrations and higher colonic aspartate concentrations [42]. Such changes were not observed following equivalent weight loss through dietary means. Changes in arginine and glutamine may also point to enhanced absorption of amino acids following RYGB. RYGB accelerates protein digestion and results in a faster and more transient post-prandial elevation in circulating amino acids [49]. In an obese Zucker rat model, RYGB was demonstrated to selectively increase glutamine absorption in biliopancreatic and Roux limbs through transcriptional upregulation of the B^0^AT1 glutamine transporter [50]. Such changes in amino acid transport may be implicated in the metabolic benefits of the procedure, as both arginine and glutamine can stimulate secretion of insulin from pancreatic β–cells, as well as GLP-1 from intestinal L-cells [51,52,53].

Reduced urinary excretion of the BCAAs valine and leucine as well as the BCAA catabolic intermediates 3-hydroxyisobutyrate (valine catabolite), 3-hydroxyisovalerate (leucine catabolite), and 3-methyl-2-oxovalerate (isoleucine catabolite) was observed following CSM. Reductions in urinary 3-hydroxyisobutyrate, 3-methyl-2-oxovalerate, and valine excretion were moderately to strongly correlated with improvements in blood pressure at months 6 and 24 in the CSM arm. Given the established links between increasing levels of BCAAs, insulin resistance, and hypertension [34,35,36,37,38,39,40], this suggests that physiological sequelae of improved BCAA catabolism in skeletal muscle and adipose tissue may play a role in the antihypertensive and metabolic benefits of RYGB [54]. Indeed, reductions in circulating BCAAs have been reported following RYGB and vertical sleeve gastrectomy [55,56], although reductions in circulating BCAAs may not be necessary to achieve the beneficial metabolic effects of obesity surgery [57].

BCAA catabolism may exert renoprotective effects by supplying tricarboxylic acid cycle intermediates when fatty acid oxidation (FAO) is impaired. Fatty acids are the primary energy source for the proximal tubule of the kidney, a highly energy-demanding region that is sensitive to injury when FAO is impaired [58]. Indeed, impairment of proximal tubular FAO is implicated in the pathogenesis of DKD [58]. Proximal tubular-specific knockdown of Krüppel-like factor 6 (KLF6), a transcription factor that is induced in the proximal tubule early in acute kidney injury and that suppresses BCAA catabolism, protected mice against acute renal injury and fibrosis [59]. Mice protected from acute kidney injury were demonstrated to have preserved expression of genes encoding BCAA catabolic enzymes [59]. Thus, enhanced BCAA catabolism may be implicated in the greater reduction in albuminuria observed following CSM at 2-year follow-up.

Our study should be interpreted in the context of certain limitations. The study size was modest, with a total of 97 samples subjected to ^1^H-NMR across two study arms (MTA and CSM) and two timepoints (baseline and month 6). Nevertheless, the fact that this sub-study was nested within one of the first RCTs to assess the impact of RYGB on a primary renal endpoint is a distinct strength of the study design [19,20]. Amongst patients included in this sub-study, there were differences in ethnicity between the two study arms, with a higher proportion of patients in the CSM arm being of Caucasian ethnicity. Similar findings were observed in the main study cohort [20]. Nevertheless, a PLS model comparing urinary ^1^H-NMR peak intensity between individuals of Caucasian (*n* = 18) and other ethnicities (*n* = 10) at baseline in the MTA arm provided no evidence of a systematic difference in urinary metabolomic profiles related to ethnicity in the present study (Figure S2). Metabolomic analyses were performed on early morning spot urine samples rather than timed urine collections. Not all samples analysed in the current study were paired samples from the same individuals at both baseline and month 6. However, we performed sensitivity analyses restricted to the subset of paired samples (nearly half of all samples) and found similar results.

PLS, as opposed to orthogonal partial least squares (OPLS), classification models were implemented in this study. While OPLS models offer the advantage of removing systematic variance in the dataset that is unrelated to the sample class [60], PLS models conducted with the R package MUVR benefit from a nested repeated double cross-validation procedure, which reduces the risk of model overfitting [61]. Contrary to conventional PLS algorithms that provide estimates from fit-predict models and cross-validation results as a point of comparison, the MUVR algorithm only provides estimates from cross-validation in order to reduce the likelihood of model overfitting and false positive discovery [61]. This is an important consideration in a study such as this with a relatively small sample size [61,62]. Unfortunately, it is not yet possible to implement OPLS classification models using the R package MUVR [61]. As such, despite the lack of assessment of variation in predictor variables orthogonal to the response, we feel that it is preferable to present results from PLS models implemented via MUVR rather than OPLS models conducted without repeated double cross-validation in the present study.

## 4. Materials and Methods

### 4.1. MOMS RCT: Study Design and Outcomes

The Microvascular Outcomes after Metabolic Surgery (MOMS) study is a single-centre, randomised, unblinded, controlled clinical trial (NCT01821508) investigating the efficacy of combined metabolic surgery plus medical therapy (CSM) versus medical therapy alone (MTA) to achieve remission of albuminuria in adults with class 1 obesity, type 2 diabetes, and microalbuminuria [20]. The main inclusion criteria were age 18–65 years, body-mass index (BMI) 30–35 kg/m^2^, type 2 diabetes with HbA_1c_ < 12%, and uACR > 30 mg/g. The primary study outcome was remission of microalbuminuria (uACR < 30 mg/g). Study recruitment occurred between 1 April 2013 and 31 March 2016.

Roux-en-Y gastric bypass (RYGB) was the metabolic surgery employed in the MOMS study, and was performed laparoscopically by a single surgeon (R.V.C.) with a 30 mL gastric pouch, 150 cm alimentary limb, and 80 cm biliopancreatic limb. Medications with beneficial effects on the microvascular and macrovascular outcomes of type 2 diabetes were administered early after trial commencement if patients were not already receiving them. Medical treatment algorithms were consistent with diabetes society guidelines that were updated during study follow-up [63]. Further details on study treatments are available in the study protocol and the 24-month outcome paper [19,20].

Study outcomes assessed at baseline as well as at months 6 and 24 and presented in this sub-study include uACR, serum creatinine, HbA_1c_, blood lipids (total cholesterol, low-density lipoprotein cholesterol (LDL-C), high-density lipoprotein cholesterol (HDL-C), and triglycerides), blood pressure, and anthropometry (body weight, waist circumference, and BMI). Blood and urine samples were obtained after a minimum 8-h fast. Spot urine samples were collected in the early morning. Laboratory measurements were performed by the same central laboratory, certified by ISO9001 and ISO14001, and accredited by the College of American Pathologists. Blood pressure was measured with a calibrated aneroid sphygmomanometer after 5 min of rest. Systolic blood pressure (SBP) and diastolic blood pressure (DBP) were calculated as the arithmetic mean of two readings obtained 5 min apart. Mean arterial blood pressure (MAP) was calculated by adding 1/3 of the pulse pressure to the DBP.

The study was conducted according to the guidelines of the Declaration of Helsinki and approved by the Institutional Research Ethics Committee of Hospital Alemão Oswaldo Cruz (study number: NCT01821508). All participants provided formal written informed consent.

### 4.2. Metabolomic Analyses: Nuclear Magnetic Resonance (NMR) Spectroscopy

The urinary metabolome was assessed by ^1^H-nuclear magnetic resonance (NMR) spectroscopy in urine samples collected at baseline and 6 months post-intervention in a subgroup of patients enrolled in the MOMS study. In total, 97 samples from both timepoints were analysed; a subset of 46 of these samples were paired samples from the same individuals at both timepoints. The number of samples analysed, stratified by study arm and timepoint, is presented in Table 4.

^1^H-NMR spectroscopy was performed according to standard Bruker In Vitro Diagnostic research (IVDr) methods [64]. Urine samples were thawed at room temperature for 20 min before a brief spin at 2000 g at 4 °C for 10 min. NMR samples for 5 mm SampleJet racks were prepared by mixing 9 parts urine with 1 part urine buffer (1.5 M KH_2_PO_4_ pD 6.95, 0.5% *w*/*v* NaN_3_, 0.1% *w*/*v* 3-trimethylsilyl propionic-2,2,3,3 acid sodium salt D4 (TSP-d4) in 99.8% D_2_O) using a Samplepro Tube L liquid handling robot (Bruker BioSpin, Ettlingen, Germany), keeping the temperature at 279 K throughout the sample preparation process. To seal the sample tubes, POM balls were added to tube caps before placing the rack in the cooled SampleJet sample changer on the spectrometer.

1D nuclear Overhauser effect spectroscopy (NOESY) and 2D J-resolved experiments were acquired for each sample with a 600 MHz Bruker Avance III HD spectrometer at 300 K equipped with a 5 mm BBI room temperature probe, using the IVDr standard parameter sets for the pulse sequences ‘noesygppr1d’ and ‘jresgpprqf’. The 1D NOESY experiment entailed collection of 32 scans into 64k data points, with a spectral width of 20 ppm, an acquisition time of 2.726 s, a relaxation delay of 4 s, a NOESY mixing time of 10 ms, 4 dummy scans at a receiver gain setting of 90.5, and presaturation offset centred on the water signal. For the 2D J-resolved experiment, after 16 dummy scans, two scans per increment were collected into 40 increments in the indirect dimension, with 8k data points in the direct dimension, and acquisition times of 0.4085 s and 0.2137 s for the direct and indirect dimensions, respectively. The relaxation delay was 2 s. The spectral window was 16.7 ppm and 93.6 Hz in the direct and indirect dimensions, respectively. The receiver gain was set to 90.5 and water suppression achieved through presaturation during the relaxation delay, similar to the 1D NOESY experiment.

Urine samples were randomised during sample preparation such that samples from different study arms and timepoints were evenly distributed during data acquisition. TSP-d4 was used for internal chemical shift referencing.

Ten pooled samples (*n* = 5 each containing aliquots of samples from both timepoints, baseline and month 6) were included as internal quality controls. The percentage coefficient of variation in PQN-normalised peak intensity amongst baseline and month 6 pooled samples was calculated for each annotated ^1^H-NMR peak (*n* = 208) as follows:

$$\left(\frac{standard\;deviation\;of\;peak\;intensity}{mean\;peak\;intensity}\right)\;\times \;100$$

Calculations were performed separately for baseline and for month 6 pooled samples, and the coefficient of variation values for each annotated peak have been uploaded to https://osf.io/vs2dg/ (accessed on 15 January 2022). A density plot highlighting the distribution of coefficient of variation values for each annotated ^1^H-NMR peak was constructed. The x-axis of the density plot was log-transformed using the ‘log1p’ transformation from the R package scales [25]. Illustrative plots of PQN-normalised spectra of pooled samples were generated for certain peaks found to be changed by CSM by multivariate analysis using the function ‘ROIplot’ from the R package speaq [26], thereby demonstrating the low variance in peak intensity between pooled samples.

To facilitate metabolite annotation, a set of 2D experiments on selected samples were acquired on an Oxford 800 MHz magnet equipped with a Bruker Avance III HD console and a 3 mm TCI cryoprobe. Multiplicity-edited ^13^C-HSQCs were acquired using the pulse sequence ‘hsqcedetgpsisp2.3’ using spectral widths of 20 and 90 ppm in the direct and indirect dimensions, respectively, collecting 64 scans per increment for a total of 512 increments and 2048 data points. The acquisition times were 63.9 and 14 ms for the direct and indirect dimensions, respectively, and the relaxation delay was 1.5 s. ^1^H-^1^H-TOCSYs were acquired using the pulse sequence ‘dipsi2esgpph’ with sweep widths in both dimensions of 12 ppm, collecting 16 scans per increment into 512 increments and 8192 data points. Acquisition times were 0.426 s and 26.6 ms for the direct and indirect dimensions, respectively. The TOCSY transfer delay was 60 ms and the relaxation delay between scans was 1 s. ^1^H-^1^H-COSYs were acquired with the pulse sequence ‘cosygpppqfpr’. Sweep widths were 13.95 ppm in both dimensions; 4 scans per increment were collected for a total of 1024 increments and 2048 data points. The acquisition time was 92 ms and the relaxation delay 2 s. All 2D spectra were referenced to TSP-d4. Tables of chemical displacements outlining ^13^C-HSQC, ^1^H-TOCSY, and ^1^H-COSY shift values for all peaks (*n* = 475) and for the subset of annotated peaks (*n* = 208) are available at: https://osf.io/vs2dg/, accessed on 15 January 2022.

### 4.3. ^1^H-NMR Spectral Processing

The 1D NOESY spectra destined for peak picking and multivariate analysis were processed by the automatic IVDr AU program, meaning they were zero-filled twice before Fourier transformation into 128k data points, adding a 0.3 Hz exponential line-broadening and referencing to TSP-d4. Spectra were processed in TopSpin3.5pl7 (Bruker BioSpin, Ettlingen, Germany). The 1D NOESY spectra were loaded into MATLAB using RBNMR [65]. Baseline correction of the spectra was performed due to the high urinary glucose concentrations present in baseline samples using the command ‘msbackadj’ with window size set to 2000, quantile set to 0.1, and step size set to 1000 [66]. The baseline-corrected 1D NOESY spectra and accompanying parts per million (ppm) chemical shift vector along with sample metadata are available at: https://osf.io/vs2dg/, accessed on 15 January 2022.

**Figure 9:**
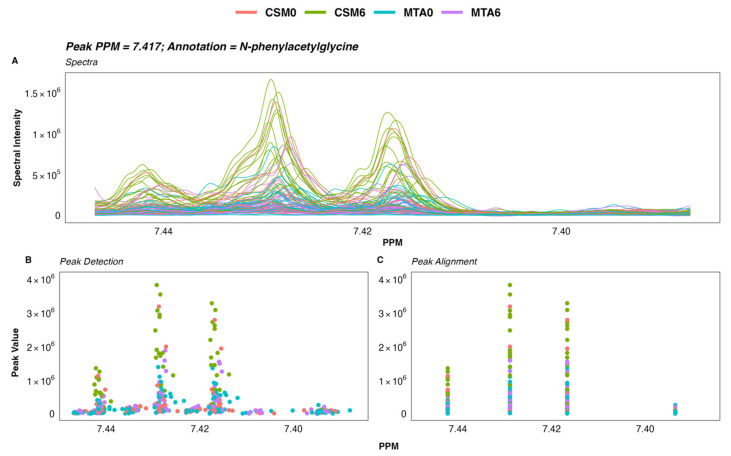
Raw spectra and spectral processing of a selected urinary ^1^H-NMR spectroscopy peak. (**A**) Raw ^1^H-NMR spectra of a selected N-phenylacetylglycine peak. Each line corresponds to the spectrum of a single sample. (**B**) Detection of peaks within the spectra using a Mexican hat wavelet transformation. Each dot represents peak intensity for a single sample. (**C**) Alignment of peaks by grouping to account for shifts in peaks between spectra due to differences in sample environment and/or experimental conditions. Each dot represents peak intensity for a single sample. Manual inspection of the spectra and spectral processing in speaq was performed as outlined here for peaks identified as important to classification performance by multivariate models to ensure that between-group differences in the identified peaks were not artefactual and could be reliably identified in the spectra. ^1^H-NMR spectroscopy, proton nuclear magnetic resonance spectroscopy; CSM, combined metabolic surgery plus medical therapy; CSM0, baseline samples from the CSM group; CSM6, month 6 samples from the CSM group; MTA, medical therapy alone; MTA0, baseline samples from the MTA group; MTA6, month 6 samples from the MTA group; PPM, parts per million chemical shift relative to TSP-d4.

Further analysis of NMR data was performed using the R statistical programming language (4.1.1) [67]. Spectra and ppm chemical shift values were imported and processed into a peak intensity matrix according to a standard workflow using the R package speaq [26]. Peak detection was performed using a Mexican hat wavelet method implemented by the function ‘getWaveletPeaks’. Detected peaks were aligned and grouped into a single ppm index value using the function ‘PeakGrouper’. Plots of the raw spectra, peak detection, and peak grouping/alignment using speaq were generated using the function ‘ROIplot’, and manually reviewed for each peak of interest. An illustrative example of one such plot is presented in Figure 9. A PDF outlining plots of the raw spectra, peak detection, peak alignment, and peak intensity by group for all annotated peaks (*n* = 208) is available at: https://osf.io/vs2dg/, accessed on 15 January 2022.

Silhouette values were calculated as a metric of the quality of peak grouping using the function ‘SilhouetR’. Peak groupings with a silhouette value less than 0.6 were removed and the peaks regrouped with the function ‘regroupR’—this process was repeated iteratively until all peak groupings had a silhouette value ≥ 0.6. Peak filling was performed to detect peaks that may have been missed during the first round of peak detection.

Finally, a peak intensity matrix was built with grouped peaks (identified by their ppm shift values) as columns and samples as rows. A probabilistic quotient normalisation (PQN) was applied to the peak intensity matrix [68,69], which was subsequently used as the input for multivariate statistics. Raw and PQN-normalised peak intensity matrices as well as sample metadata are available at: https://osf.io/vs2dg/, accessed on 15 January 2022. Annotation of processed spectra was performed using Chenomx 8.6 software (Chenomx Inc.), the Human Metabolome Database (HMDB; https://hmdb.ca/; accessed on 15 January 2022), the Biological Magnetic Resonance Data Bank (BMRB; https://bmrb.io/; accessed on 15 January 2022), and the acquired 2D NMR data for selected samples [70,71,72].

### 4.4. Principal Component Analysis of ^1^H-NMR Data

Clustering by principal component analysis (PCA) was performed on PQN-normalised NMR peak intensities using the function ‘prcomp’, and plotted by phenotype along principal components 1-3 using the function ‘scatter3D’ from the R package plot3D [73]. Baseline samples from the CSM and MTA arms were considered together in order to increase clarity on the PCA plot. Centroids were calculated as the mean scores along principal components 1–3 for the three groups: baseline, MTA6, and CSM6. Ellipsoids were calculated along principal components 1–3 using the function ‘makeEllipsoid’ from the R package ChemoSpec, and plotted using ‘scatter3D’ [27,73]. Loadings were extracted from the principal component object and plotted using the function ‘scatter3D’, with selected peaks labelled [73].

### 4.5. Fold Change Testing of ^1^H-NMR Data

For between-group comparisons of interest, fold change in PQN-normalised urinary ^1^H-NMR peak intensity was calculated. For between-group comparisons using all available samples, unpaired *t*-tests were performed using the R package rstatix to compare intensity of each peak between the two groups of samples [74]. For between-group comparisons using only paired samples, paired *t*-tests were performed using the R package rstatix [74]. *p*-values were multiplicity corrected for the number of comparisons (peaks) using the Benjamini–Hochberg method [75]. For between-group comparisons using all available samples, mean peak intensity for each group was extracted from the unpaired *t*-test object. For between-group comparisons of paired samples, mean peak intensity by group was calculated using the base R function ‘mean’ as mean estimates were not returned in the paired *t*-test objects [67]. Log2 fold change was calculated as:

$$log2\left(\frac{mean\;peak\;intensity\;of\;group\;2}{mean\;peak\;intensity\;of\;group\;1}\right)$$

Results of fold change testing for comparisons using all available samples (MTA0 vs. CSM0, MTA0 vs. MTA6, CSM0 vs. CSM6, and MTA6 vs. CSM6) are presented as volcano plots, with log2 fold change on the *x*-axis and –log10 of the multiplicity-corrected *p*-value on the *y*-axis. Log2 fold change values and associated *p*-values for the CSM0 vs. CSM6 all available samples, CSM0 vs. CSM6 paired samples, and MTA6 vs. CSM6 comparisons are also summarized in a table for peaks identified as changed by CSM from baseline to month 6 by multivariate partial least squares (PLS) classification models.

### 4.6. ^1^H-NMR Classification Modelling

To elucidate differences in the urinary metabolome between patients treated with CSM and MTA, the R package MUVR was used to fit a series of multivariate PLS classification models for comparison of two groups of samples at a time [61]. The MUVR models were fitted to PQN-normalised ^1^H-NMR peak intensity matrices (X matrices) containing the relevant samples for each comparison [61]. A Y response vector indicating sample group assignment was inputted to each supervised model.

The groups of samples utilised for comparisons interrogated by MUVR models are outlined in Table 5, along with the number of samples inputted to each model as well as the location of model results within the manuscript. As outlined in Table 4 above, not all samples from baseline to month 6 were necessarily from the same individuals. In order to maximize sample usage as well as statistical power, the primary PLS models were run on all available samples (a mixture of unpaired and paired samples) for a given comparison. As sensitivity analyses, PLS models were also fitted to the subset of paired samples from the same individuals at both timepoints for relevant comparisons. Sample IDs were inputted to these paired sample models in an effort to reduce model overfitting by ensuring that samples from the same individual were kept together in cross-validation segments.

The MUVR algorithm minimises overfitting in multivariate modelling by performing recursive elimination of the least informative variables in a repeated double cross-validation procedure [61]. The following modelling parameters were used as recommended: nOuter = 8 (number of outer cross-validation segments, to ensure both classes were present in all model segments), nRep = 100 (number of model repetitions, high number was selected to improve reproducibility), and varRatio = 0.85 (proportion of variables maintained in the data per model iteration during variable elimination) [61].

MUVR returns three consensus models (‘min’, ‘mid’, and ‘max’) with similar fitness [61]. The ‘max’ model, which considers all relevant predictors without compromising classification performance, was selected to identify as many urinary ^1^H-NMR peaks as possible that were relevant to classifying urine samples according to group assignment. Model stability across 100 repetitions was ensured by inspecting the number and proportion of selected variables as well as the number of misclassifications per repetition and cumulatively; convergence occurred by 20 repetitions for all models.

The area under the receiver operating characteristic curve (AUC) and number of model misclassifications was used to assess model performance. The number of misclassifications is used to assess fitness of MUVR PLS classification models, rather than metrics such as R2X, R2Y, and Q2 [61]. AUC and the associated 95% confidence interval were calculated by inputting actual sample class alongside the predicted sample class probability (obtained from the MUVR model) to the function ‘roc’ from the R package pROC [76]. Receiver operating characteristic (ROC) curves were generated using the function ‘ggroc’ from the pROC package [76].

Additional performance metrics relating to model classification, including overall classification accuracy, the Kappa statistic, sensitivity, specificity, positive predictive value, and negative predictive value, were calculated by inputting actual sample class alongside predicted sample class at the 50% probability threshold (obtained from the MUVR model) to the function ‘confusionMatrix’ from the R package caret [77]. These performance metrics are presented in a table for each model, prepared using the function ‘ggtexttable’ from the R package ggpubr [78]. A confusion matrix was plotted for each MUVR model outlining the number and proportion of correct and incorrect model classifications using a custom ggplot2 plotting function [79].

The MUVR function ‘plotVAL’ was used to generate plots of model validation performance [61], which were inspected to ensure that the validation performance was optimised (in other words, the number of misclassifications was minimised) after recursive variable elimination to produce the MUVR ‘max’ model for each comparison. It is worth noting that the absolute number of misclassifications during model training and tuning in the inner segments differs from the final number of model misclassifications outlined in confusion matrices as there are multiple outer and inner segments per MUVR model as part of the repeated double cross-validation procedure [61]. Additionally, data is reused in the inner segments, which may result in an overestimation of model performance at this stage of the MUVR algorithm [61].

Permutation testing was also performed using the R package MUVR to further evaluate modelling performance [61]. Permutations were performed for each MUVR model by repeated random sampling (using the base R function ‘sample’) of the Y response vector indicating sample class. Thereafter, the original X matrix of PQN-normalised ^1^H-NMR peak intensity was modelled on permuted Y responses. A total of 500 permutations was performed for each MUVR model evaluating a given between-group comparison. Permutation modelling parameters were the same as those used for the final MUVR models outlined above (nOuter = 8, varRatio = 0.85), with the exception of the number of model repetitions, which was reduced to 25 per permuted model due to computational restraints. Authors of the MUVR package recommend performing ≥ 20–50 model repetitions for final processing of actual models with the correct Y response vector [61]. They also note that compromises may need to be made on the number of model repetitions during permutation testing in order to reduce computational cost, and that results from models with slightly different parameter settings usually correlate strongly [61]. Thus, estimates obtained from permuted models should be reliable.

Results of permutation testing are presented as histograms and density curves outlining the distribution of misclassifications by permuted models, with results from the actual model based on the correct Y response vector superimposed. After ensuring the presence of a Gaussian distribution of permuted model misclassifications, a parametric Student’s *t*-test was performed using the MUVR function ‘pPerm’ to establish the statistical significance of differences in misclassification rates between the actual and permuted models [61].

Variable importance in projection (VIP) was used to rank variable (urinary ^1^H-NMR peak) importance to PLS models [61]. Dotplots of ^1^H-NMR peaks ordered by VIP rank to PLS models were generated using custom ggplot2 plotting functions [79]. In order to improve the reproducibility of model findings, VIP values presented are the mean of estimates obtained from 100 model repetitions. Unannotated peaks were removed from these plots for clarity. Heatmaps are presented adjacent to dotplots of peaks ranked by VIP to indicate the relative abundance of individual peaks between the two groups compared in PLS models. Mean PQN-normalised peak intensity was calculated across all samples in the two groups evaluated in a given PLS model. Mean peak intensity values by group were subsequently centred and scaled using the function ‘scale_rows’ from the R package pheatmap, and plotted on heatmaps to indicate mean directionality of peak change between the two groups. The y-axis of these heatmaps also provides the ppm chemical shift value corresponding to annotated peaks in dotplots of peaks ranked by VIP.

### 4.7. Inter-Peak Correlations

Pearson correlations between urinary ^1^H-NMR peaks were performed by creating a correlation matrix from PQN-normalised peak intensity values for selected metabolites of interest using the base R function ‘cor’ [67]. Metabolites that were strongly changed by CSM from baseline to month 6 were selected. Metabolites were primarily selected on the basis of their importance to classification of CSM6 samples in multivariate PLS models. Further metabolites were manually selected following review of the ^1^H-NMR spectra and on the basis of belonging to similar functional groupings to those identified by PLS models. Inter-peak correlations were clustered and plotted on a heatmap using the R package pheatmap [80].

### 4.8. Correlations between Changes in Urinary Metabolites with Changes in Metabolic and Renal Indices Following CSM

The relationships between changes in PQN-normalised urinary ^1^H-NMR peak abundance from baseline to month 6 with changes in metabolic and renal indices from baseline to month 6 and from baseline to month 24 were investigated by constructing a correlation matrix and performing pairwise Pearson correlations using the base R function ‘cor’ [67]. The *p*-values of correlations were computed using the function ‘cor_pmat’ from the R package ggcorrplot [81]. Correlation analysis was performed for the subset of individuals in the CSM arm with paired urinary ^1^H-NMR peak data available at baseline and month 6 (*n* = 11). Pearson r values between 0.5 and 0.7 were considered moderately strong while those > 0.7 were considered strong. *p*-values of correlation < 0.05 were considered statistically significant. However, given the modest sample size that limited statistical power, *p*-values of correlation ≥ 0.05 may still reflect biologically meaningful relationships that did not achieve statistical significance in these exploratory analyses.

Metabolites reflective of host–microbial co-metabolism and BCAA catabolism were selected for correlations as these two groups of metabolites were strongly changed by CSM from baseline to month 6. Absolute difference from baseline to follow-up in urinary metabolites and in clinical parameters was calculated prior to correlation analysis. uACR values were natural logarithm-transformed. Two correlation matrices, one outlining the Pearson r correlation values and the other presenting the *p*-values of correlations, were plotted and customised using ggplot2 [79].

To contrast differences in relationships between early changes in BMI and early changes in urinary metabolites reflective of host–microbial co-metabolism and BCAA catabolism with improvements in metabolic and renal indices following CSM, correlation scatterplots were constructed. Scatterplots of absolute difference in clinical parameters (MAP, HbA_1c_, triglycerides, and log uACR) with absolute difference in urinary peak abundance and in BMI were created [79], with Pearson correlation coefficients and *p*-values of correlations added using the function ‘stat_cor’ from the R package ggpubr [78]. Plots were facetted by the metabolic and renal indices included in the correlation analysis (MAP, HbA_1c_, triglycerides, and log uACR) as well as timing of clinical parameter assessment (month 6 vs. month 24).

### 4.9. Descriptive and Inferential Statistics

Baseline clinical parameters were summarized by study arm as *n* (%) for categorical variables, mean ± standard deviation for normally distributed continuous variables, and median [interquartile range] for continuous variables that were not normally distributed. χ^2^ analysis was used to analyse for differences in categorical variables between the CSM and MTA arms, except where the frequency of values was < 5 in at least 1 cell of the contingency table, in which case Fisher’s exact test was used. Normally distributed continuous variables were compared between the CSM and MTA arms by unpaired *t*-tests, while skewed continuous variables were compared between groups by Wilcoxon rank sum tests.

Absolute and percentage differences in clinical parameters from baseline to follow-up (months 6 and 24) were calculated for the sub-study cohort with available paired urine samples. Absolute and percentage differences were summarized by study arm as median [interquartile range] and compared between groups by Wilcoxon rank sum tests.

Statistical significance of changes in urinary ^1^H-NMR peak abundance in the CSM and MTA arms from baseline to month 6 was evaluated by unpaired *t*-tests. *p*-values were multiplicity-corrected for the number of comparisons using the Benjamini–Hochberg method and presented on ggplot2-rendered plots of peak abundance by study arm and timepoint using the function ‘stat_pvalue_manual’ from the R package ggpubr [75,78,79].

Statistical analyses were performed using the R package rstatix in RStudio (R version 4.1.1) [67,74]. *p* < 0.05 was considered statistically significant.

## 5. Conclusions

Changes in the urinary metabolome following MTA were less distinctive than those occurring following CSM. Urinary excretion of the host–microbial co-metabolites N-phenylacetylglycine, trimethylamine N-oxide, and 4-aminobutyrate (GABA) as well as the amino acids arginine and glutamine increased following CSM. Reduced urinary excretion of aromatic amino acids (phenylalanine and tyrosine) as well as BCAAs and related catabolites also occurred following CSM. Early changes in BMI following CSM were not meaningfully associated with the sustained improvements in metabolic and renal indices observed following the intervention. Importantly, early (month 6) changes in urinary metabolites reflective of host–microbial co-metabolism and BCAA catabolism following CSM were moderately to strongly correlated with improvements in clinical indices of adverse metabolic and renal health at months 6 and 24. In summary, our findings implicate RYGB-induced urinary metabolomic changes in host–microbial co-metabolism, aromatic amino acid metabolism, and BCAA catabolism in the enhanced improvements in blood pressure, glycaemia, dyslipidaemia, and albuminuria observed in patients with obesity, type 2 diabetes, and early CKD treated with CSM in the MOMS RCT.

## Figures and Tables

**Table 1:** Baseline characteristics of the sub-study cohort ^1,2^.

Variable	CSM (*n* = 26)	MTA (*n* = 28)	*p*
**Demographics**			
Age (mean ± SD; years)	51.8 ± 8.1	50.3 ± 7.4	0.50
Male (*n* (%))	15 (57.7)	13 (46.4)	0.58
Caucasian ethnicity (*n* (%))	24 (92.3)	18 (64.3)	0.05
**Anthropometry**			
Body weight (kg)	94.5 ± 14.0	91.6 ± 14.5	0.45
Waist circumference (cm)	114.3 ± 7.9	112.0 ± 8.4	0.33
Body-mass index (mean ± SD; kg/m^2^)	33.0 ± 2.0	32.8 ± 2.1	0.75
**Blood pressure**			
Systolic blood pressure (mean ± SD; mmHg)	143.8 ± 20.2	138.9 ± 13.7	0.32
Diastolic blood pressure (mean ± SD; mmHg)	91.6 ± 14.5	86.4 ± 7.8	0.13
**Metabolic parameters**			
Total cholesterol (mean ± SD; mg/dL)	182.2 ± 38.2	204.5 ± 45.6	0.06
LDL cholesterol (mean ± SD; mg/dL)	102.9 ± 37.3	120.2 ± 41.9	0.12
HDL cholesterol (mean ± SD; mg/dL)	37.3 ± 9.4	38.8 ± 9.2	0.54
Triglycerides (median [IQR]; mg/dL)	189.5 [147.0]	208.5 [199.3]	0.43
HbA_1c_ (mean ± SD; mmol/mol)	74.2 ± 23.1	73.9 ± 18.0	0.95
HbA_1c_ (mean ± SD; %)	8.9 ± 2.1	8.9 ± 1.6	0.95
**Renal parameters**			
Serum creatinine (mean ± SD; mg/dL)	0.79 ± 0.22	0.82 ± 0.29	0.72
uACR (median [IQR]; mg/g)	70.0 [43.8]	73.5 [97.8]	0.46

^1^ CSM, combined metabolic surgery plus medical therapy; HbA_1c_, glycated haemoglobin; HDL, high-density lipoprotein; IQR, interquartile range; LDL, low-density lipoprotein; MTA, medical therapy alone; SD, standard deviation; uACR, urinary albumin-to-creatinine ratio. ^2^ Values are given as *n* (%) for categorical variables, mean ± SD for normally distributed continuous variables, and median [IQR] for continuous variables that are not normally distributed. χ^2^ analysis was used to analyse for between-group differences in categorical variables, except where the frequency of values was <5 in at least one cell of the contingency table, in which case, Fisher’s exact test was used. Normally distributed continuous variables were compared between groups by unpaired *t*-tests, while skewed continuous variables were compared between groups by Wilcoxon rank sum tests.

**Table 2:** Changes in study outcomes at months 6 and 24 in the sub-study cohort with available paired urine samples ^1,2^.

	Timepoint	Absolute Difference			Percentage Difference		
		CSM (*n* = 11)	MTA (*n* = 12)	*p*	CSM (*n* = 11)	MTA (*n* = 12)	*p*
Body-mass index (median [IQR]; kg/m^2^ [absolute] or %)	Month 6	−9.0 [1.0]	−1.0 [2.3]	**<0.001**	−25.7 [4.6]	−2.9 [6.4]	**<0.001**
	Month 24	−9.0 [1.8]	−2.0 [2.5]	**<0.001**	−25.8 [2.9]	−5.7 [7.9]	**<0.001**
Systolic blood pressure (median [IQR]; mmHg [absolute] or %)	Month 6	−20.0 [15.0]	−5.0 [10.0]	**0.01**	−14.3 [7.7]	−3.3 [7.3]	**0.005**
	Month 24	0 [28.0]	0 [25.0]	0.94	0 [17.5]	0 [17.1]	0.94
Diastolic blood pressure (median [IQR]; mmHg [absolute] or %)	Month 6	−15.0 [17.5]	0 [10.0]	0.19	−15.6 [18.9]	0 [10.3]	0.18
	Month 24	−10.0 [10.0]	0 [10.0]	0.28	−10.0 [11.1]	0 [11.3]	0.23
LDL-cholesterol (median [IQR]; mg/dL [absolute] or %)	Month 6	−14.0 [41.5]	−14.0 [27.0]	0.76	−14.0 [33.4]	−8.4 [23.9]	0.57
	Month 24	−21.0 [42.3]	−13.0 [66.5]	0.53	−17.2 [42.6]	−10.7 [50.9]	0.31
Triglycerides (median [IQR]; mg/dL [absolute] or %)	Month 6	−86.0 [107.5]	−40.0 [85.5]	0.42	−53.7 [40.9]	−19.8 [27.6]	0.10
	Month 24	−68.0 [74.5]	−27.0 [117.5]	0.28	−38.7 [34.8]	−11.5 [52.8]	0.15
HbA_1c_ (median [IQR]; mmol/mol [absolute] or %)	Month 6	−39.3 [37.2]	−9.8 [15.3]	**0.02**	−43.9 [34.8]	−15.1 [18.0]	**0.009**
	Month 24	−33.9 [28.4]	−12.0 [17.5]	0.11	−36.5 [32.3]	−27.8 [19.3]	0.13
uACR (median [IQR]; mg/g [absolute] or %)	Month 6	−67.0 [35.0]	−51.0 [92.3]	0.95	−80.6 [16.7]	−70.4 [32.2]	0.29
	Month 24	−45.5 [41.0]	−49.0 [100.0]	1	−90.1 [29.0]	−84.6 [12.9]	0.58

^1^ CSM, combined metabolic surgery plus medical therapy; HbA_1c_, glycated haemoglobin; IQR, interquartile range; LDL, low-density lipoprotein; MTA, medical therapy alone; uACR, urinary albumin-to-creatinine ratio. ^2^ Values are given as median [IQR]. Between-group comparisons were made by Wilcoxon rank sum tests. *p*-values < 0.05 are bolded.

**Table 3:** Summary of the top urinary ^1^H-NMR peaks identified by CSM0 vs. CSM6 and MTA6 vs. CSM6 PLS classification models ^1,2,3^.

Metabolite(s)	PPM	HMDB ID	CSM6 Abundance	All Available Samples CSM0 (*n* = 26) vs. CSM6 (*n* = 19)			Paired Samples CSM0 (*n* = 11) vs. CSM6 (*n* = 11)			All Available Samples MTA6 (*n* = 24) vs. CSM6 (*n* = 19)		
				log2FC	p.adj	VIP	log2FC	p.adj	VIP	log2FC	p.adj	VIP
N-phenylacetyl-glycine	7.366	0000821	↑	1.81	<0.001	4.87	2.38	0.007	4.21	1.37	0.01	5.47
Glutamine + glutamate	2.098	0000641 + 0000148	↑	1.11	0.001	11.77	1.41	0.007	11.83	0.81	0.02	9.19
Glycerol-3-phosphate + ethanol	3.659	0000126 + 0000108	↑	1.25	0.002	17.89	1.57	0.02	32.48	0.83	0.04	31.21
Glutamine	2.143	0000641	↑	1.13	0.002	21.00	1.16	0.03	79.40	0.97	0.02	20.05
Arginine	1.948	0000517	↑	1.32	0.004	22.27	1.59	0.02	37.72	1.10	0.02	13.85
Not annotated + S-sulfocysteine + pyroglutamate	4.185	N/A + 0000731 + 0000267	↑	1.02	0.003	22.28	1.37	0.02	46.82	0.82	0.02	14.72
4-aminobutyrate	2.287	0000112	↑	1.73	0.007	26.15	2.19	0.04	66.71	1.45	0.03	18.26
Trimethylamine N-oxide	3.278	0000925	↑	1.38	0.003	33.63	1.78	0.02	43.75	0.94	0.04	35.38
Glucose	5.348	0000122	↓	−0.69	0.03	34.48	−0.61	0.28	231.23	-0.86	0.03	20.34
Valine	1.052	0000883	↓	−0.47	0.003	36.53	−0.59	0.03	67.48	−0.47	0.04	43.10
Methylguanidine	2.836	0001522	↑	0.64	0.005	45.90	1.02	0.02	43.52	0.31	0.39	292.93
Phenylalanine	7.394	0000159	↓	−1.74	0.01	78.94	−3.10	0.02	41.33	−1.86	0.05	59.63
Urea + xanthosine	5.861	0000294 + 0000299	↑	0.45	0.04	88.00	0.86	0.02	38.63	0.09	0.67	N/A

^1^ CSM0, baseline samples from CSM group; CSM6, month 6 samples from CSM group; HMDB, Human Metabolome Database; log2FC, log2 fold change; MTA6, month 6 samples from MTA group; N/A, not applicable; p.adj, adjusted *p*-value (Benjamini–Hochberg multiplicity correction); PLS, partial least squares; PPM, parts per million chemical shift relative to TSP-d4; VIP, variable importance in projection. ^2^ Log2 fold change values and adjusted *p*-values (Benjamini–Hochberg) were obtained from univariate testing of between-group differences in urinary ^1^H-NMR peak intensity by unpaired *t*-tests. Log2 fold change values and adjusted *p*-values derived from univariate testing of the CSM0 vs. CSM6 (all available samples) and MTA6 vs. CSM6 (all available samples) comparisons are also outlined in the volcano plots in Figure 3, panels C and D. Variable importance in projection (VIP) values were obtained from multivariate PLS classification models for between-group comparisons. ^3^ Where multiple metabolites are present in a given peak, metabolites are listed in order of relative abundance in the peak, with the most abundant metabolite listed first.

**Table 4:** Overview of urine samples analysed by ^1^H-NMR in the current study, stratified by study arm and timepoint ^1^.

Sample Number	Breakdown by Study Arm	Breakdown by Study Timepoint	Breakdown by Study Group and Timepoint
**All samples**			
*n* = 97	CSM, *n* = 45	Baseline, *n* = 54	CSM0, *n* = 26
	MTA, *n* = 52	Month 6, *n* = 43	CSM6, *n* = 19
			MTA0, *n* = 28
			MTA6, *n* = 24
**Paired samples**			
*n* = 46	CSM, *n* = 22	Baseline, *n* = 23	CSM0, *n* = 11
	MTA, *n* = 24	Month 6, *n* = 23	CSM6, *n* = 11
			MTA0, *n* = 12
			MTA6, *n* = 12

^1^ CSM, combined metabolic surgery plus medical therapy; CSM0, baseline samples from CSM group; CSM6, month 6 samples from CSM group; MTA, medical therapy alone; MTA0, baseline samples from MTA group; MTA6, month 6 samples from MTA group.

**Table 5:** Overview of MUVR models and location of results within the manuscript ^1^.

Comparison	Number of Samples	Figure Number
**All samples (*n* = 97 in total; primary analyses)**		
MTA0 vs. CSM0	Total, *n* = 54 CSM0, *n* = 26 MTA0, *n* = 28	Figure S1
Caucasian vs. other ethnicities in MTA0 samples	Total, *n* = 28 Caucasian, *n* = 18 Other ethnicities, *n* = 10	Figure S2
MTA0 vs. MTA6	Total, *n* = 52 MTA0, *n* = 28 MTA6, *n* = 24	Figure S3
CSM0 vs. CSM6	Total, *n* = 45 CSM0, *n* = 26 CSM6, *n* = 19	Figure 4
MTA6 vs. CSM6	Total, *n* = 43 CSM6, *n* = 19 MTA6, *n* = 24	Figure 5
**Paired samples (*n* = 46 in total; sensitivity analyses)**		
MTA0 vs. MTA6	Total, *n* = 24 MTA0, *n* = 12 MTA6, *n* = 12	Figure S4
CSM0 vs. CSM6	Total, *n* = 22 CSM0, *n* = 11 CSM6, *n* = 11	Figure S5

^1^ CSM, combined metabolic surgery plus medical therapy; CSM0, baseline samples from CSM group; CSM6, month 6 samples from CSM group; MTA, medical therapy alone; MTA0, baseline samples from MTA group; MTA6, month 6 samples from MTA group; MUVR, multivariate methods with unbiased variable selection in R.

## Data Availability

The urinary NMR spectroscopy data presented in this manuscript has been uploaded to the Open Science Framework at https://osf.io/vs2dg/, accessed on 15 January 2022. Uploaded data include sample metadata, raw and PQN-normalised 1D NOESY spectra, a parts per million (ppm) chemical shift vector to accompany the 1D NOESY spectra, raw and PQN-normalised processed peak intensity matrices, and tables of chemical displacements outlining ^13^C-HSQC, ^1^H-TOCSY, and ^1^H-COSY shift values for all peaks (*n* = 475) and for the subset of annotated peaks (*n* = 208). A PDF outlining spectral processing as well as peak abundance by study arm and timepoint for each annotated peak in the urinary ^1^H-NMR spectra has also been uploaded to the Open Science Framework repository. Percentage coefficient of variation values in PQN-normalised peak intensity amongst baseline and month 6 pooled samples for each annotated peak have also been uploaded to the repository. All remaining data presented in this study are available from the authors upon written request and following agreement on the intended purpose of the request.

## Supplementary Materials

The following are available online at https://www.mdpi.com/article/10.3390/metabo12020139/s1, Figure S1: Multivariate analysis of between-group differences in the urinary metabolome at baseline (all available samples); Figure S2: Multivariate analysis of ethnicity-related differences in the urinary metabolome at baseline in the MTA arm (all available samples); Figure S3: Multivariate analysis of changes in the urinary metabolome from baseline to month 6 in the MTA arm (all available samples); Figure S4: Multivariate analysis of changes in the urinary metabolome from baseline to month 6 in the MTA arm (paired samples only); Figure S5: Multivariate analysis of changes in the urinary metabolome from baseline to month 6 in the CSM arm (paired samples only); Figure S6: Matrix of *p*-values for Pearson correlations between changes in clinical parameters at months 6 and 24 with changes in urinary metabolites reflective of host–microbial co-metabolism and BCAA catabolism at month 6; Figure S7: Scatterplots of month 6 changes in BMI and in urinary host–microbial co-metabolites with changes in blood pressure, glycaemia, lipids, and uACR in the CSM arm during follow-up; Figure S8: Scatterplots of month 6 changes in BMI and in urinary metabolites reflective of BCAA catabolism with changes in blood pressure, glycaemia, lipids, and uACR in the CSM arm during follow-up.
