# Supplementary material for: Urinary Metabolomic Changes Accompanying Albuminuria Remission following Gastric Bypass Surgery for Type 2 Diabetic Kidney Disease
Source: Metabolites. Author manuscript; Available in PMC 2022 Feb 19. (PMC7612403; doi:10.3390/metabo12020139)
Supplement: Supplementary file 1 [file EMS142304-supplement-Supplementary_material.pdf]

Supplementary Material

Urinary Metabolomic Changes Accompanying Albuminuria Remission following Gastric Bypass Surgery for Type 2 Diabetic Kidney Disease

William P. Martin [1], Daniel Malmodin [2], Anders Pedersen [2], Martina Wallace [3], Lars Fändriks [4], Cristina M. Aboud [5], Tarissa B. Zanata Petry [5], Lívia P. Cunha da Silveira [5], Ana C. Calmon da Costa Silva [5], Ricardo V. Cohen [5], Carel W. le Roux [1,6,†] and Neil G. Docherty [1,*,†]

1 Diabetes Complications Research Centre, School of Medicine, Conway Institute of Biomolecular and Biomedical Research, University College Dublin, Belfield, Dublin 4, Ireland; william.martin@ucd.ie (W.P.M.); carel.leroux@ucd.ie (C.W.l.R.); neil.docherty@ucd.ie (N.G.D.)
2 Swedish NMR Centre, University of Gothenburg, Sweden; daniel.malmodin@nmr.gu.se (D.M.); anders.pedersen@nmr.gu.se (A.P.)
3 Institute of Food and Health, School of Agriculture and Food Science, University College Dublin, Belfield, Dublin 4, Ireland; martina.wallace@ucd.ie (M.W.)
4 Institute of Clinical Sciences, Sahlgrenska Academy, University of Gothenburg, Sweden; lars.fandriks@gastro.gu.se (L.F.)
5 The Centre for Obesity and Diabetes, Oswaldo Cruz German Hospital, São Paulo, Brazil; cmamedio@haoc.com.br (C.M.A.); tpetry@haoc.com.br (T.B.Z.P.); lpcunha@haoc.com.br (L.P.C.d.S.); calmon81@hotmail.com (A.C.C.d.C.S.); ricardo.cohen@haoc.com.br (R.V.C.)
6 Diabetes Research Group, Ulster University, Coleraine, United Kingdom
* Correspondence: neil.docherty@ucd.ie
† These two authors have contributed equally to this work and share senior authorship.

Table of Contents:

Figure S1. Multivariate analysis of between-group differences in the urinary metabolome at baseline (all available samples).

Figure S2. Multivariate analysis of ethnicity-related differences in the urinary metabolome at baseline in the MTA arm (all available samples).

Figure S3. Multivariate analysis of changes in urinary metabolome from baseline to month 6 in the MTA arm (all available samples).

Figure S4. Multivariate analysis of changes in the urinary metabolome from baseline to month 6 in the MTA arm (paired samples only).

Figure S5. Multivariate analysis of changes in the urinary metabolome from baseline to month 6 in the CSM arm (paired samples only).

Figure S6. Matrix of p-values for Pearson correlations between changes in clinical parameters at months 6 and 24 with changes in urinary metabolites reflective of host-microbial co-metabolism and BCAA catabolism at month 6.

Figure S7. Scatterplots of month 6 changes in BMI and in urinary host-microbial co-metabolites with changes in blood pressure, glycaemia, lipids, and uACR in the CSM arm during follow-up.

Figure S8. Scatterplots of month 6 changes in BMI and in urinary metabolites reflective of BCAA catabolism with changes in blood pressure, glycaemia, lipids, and uACR in the CSM arm during follow-up.

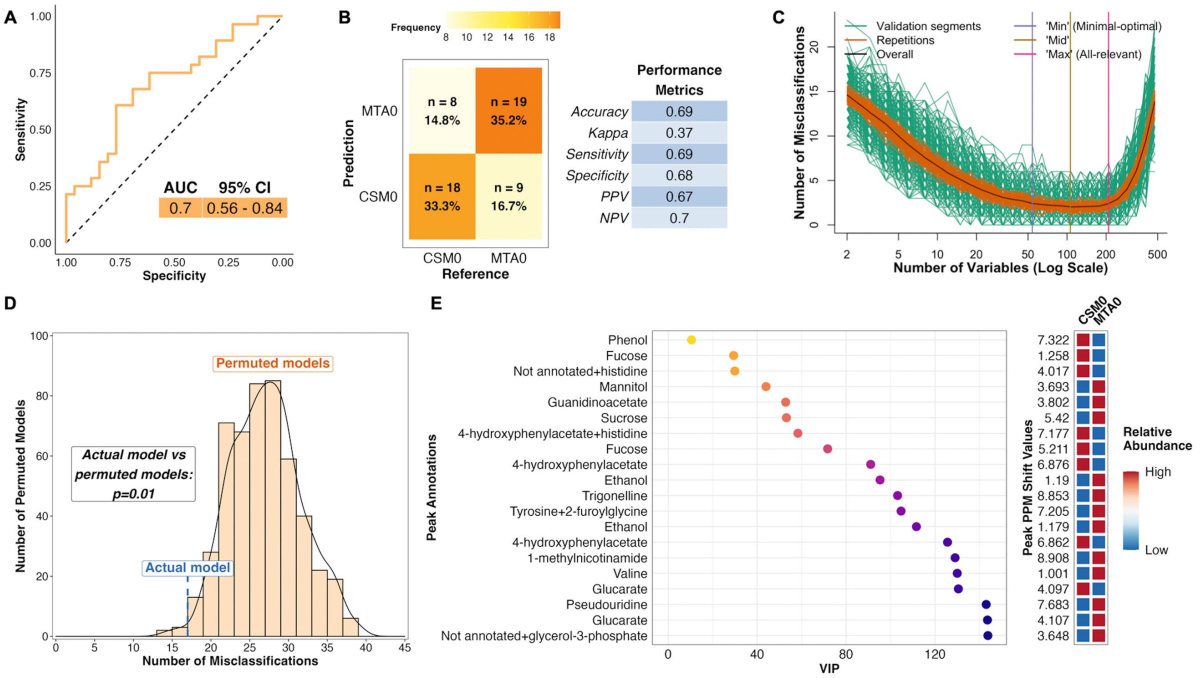

Figure S1. Multivariate analysis of between-group differences in the urinary metabolome at baseline (all available samples). Number of samples inputted to the MUVR PLS model: CSM0, n=26; MTA0, n=28. **(a)** Receiver operating characteristic curve for the MUVR PLS classification model fit to urinary ^{1}H-NMR peaks for these two groups of samples. Inset are the AUC and associated 95% CI for the curve. **(b)** Confusion matrix outlining sample classification by the PLS model. The x-axis presents the actual class of the inputted samples. The y-axis presents the sample class predicted by the models. Correctly and incorrectly classified samples are outlined on opposing diagonals of the confusion matrix. Additional performance metrics relating to sample classification at the 50% probability threshold are presented in the table adjacent to the confusion matrix. **(c)** Recursive ranking and backward elimination of variables (right-to-left on the x-axis) in the inner segments of the MUVR PLS model to achieve optimal validation performance, quantified by the number of misclassifications (y-axis). Green lines represent validation curves per inner segment and may fluctuate. Orange and black lines represent inner segment curves averaged per model repetition and overall (100 model repetitions), respectively, and describe the actual validation performance at higher resolution. Vertical lines outline the number of variables selected in MUVR 'min', 'mid', and 'max' models on the x-axis. **(d)** Histogram and density curve outlining the distribution of misclassifications by 500 permuted PLS models in which the Y response vector indicating sample class was randomly sampled. The number of misclassifications by the actual PLS model with the correct Y response vector is outlined on the x-axis by the blue dashed line. A p-value derived from a student's t-test comparing the number of misclassifications between actual and permuted models is presented adjacent to the histogram and density curve. **(e)** Dotplot of the top 20 most important annotated peaks to performance of the PLS model ranked by VIP values. Multiple peaks were identified for certain metabolites. When multiple metabolites are present in a given peak, metabolites are listed in order of relative abundance in the peak, with the most abundant metabolite listed first. The adjacent heatmap illustrates relative abundance of metabolites based on mean peak intensity across all samples in the two groups evaluated by the PLS model. 95% CI, 95% confidence interval; AUC, area under the receiver operating characteristic curve; CSM0, baseline samples from CSM group; MTA0, baseline samples from MTA group; MUVR, multivariate methods with unbiased variable selection in R; NMR, nuclear magnetic resonance spectroscopy; NPV, negative predictive value; PLS, partial least squares; PPM, parts per million chemical shift relative to TSP-d4; PPV, positive predictive value; VIP, variable importance in projection.

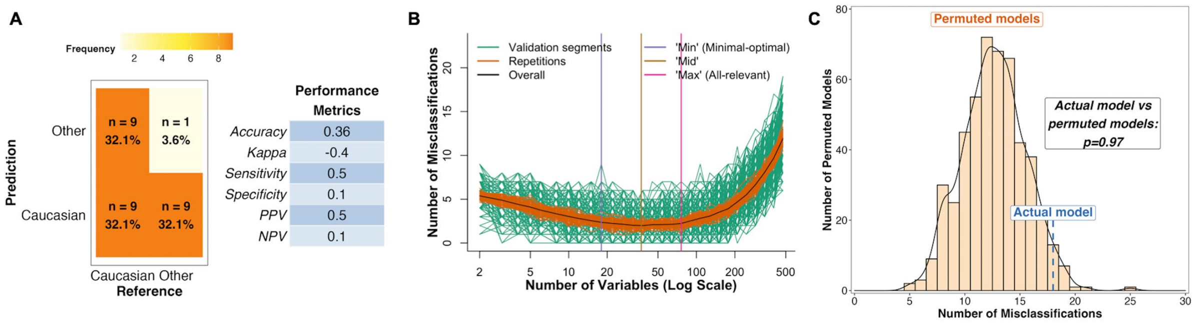

Figure S2. Multivariate analysis of ethnicity-related differences in the urinary metabolome at baseline in the MTA arm (all available samples). Number of samples inputted to the MUVR PLS model: Caucasian, n=18; other ethnicities, n=10. **(a)** Confusion matrix outlining sample classification by the PLS model. The x-axis presents the actual class of the inputted samples. The y-axis presents the sample class predicted by the models. Correctly and incorrectly classified samples are outlined on opposing diagonals of the confusion matrix. Additional performance metrics relating to sample classification at the 50% probability threshold are presented in the table adjacent to the confusion matrix. **(b)** Recursive ranking and backward elimination of variables (right-to-left on the x-axis) in the inner segments of the MUVR PLS model to achieve optimal validation performance, quantified by the number of misclassifications (y-axis). The absolute number of misclassifications during model training and tuning in the inner segments differs from the final number of model misclassifications outlined in the confusion matrix as there are multiple outer and inner segments per MUVR model as part of the repeated double cross-validation procedure. Additionally, data is reused in the inner segments, which may result in an overestimation of model performance at this stage of the MUVR algorithm. Green lines represent validation curves per inner segment and may fluctuate. Orange and black lines represent inner segment curves averaged per model repetition and overall (100 model repetitions), respectively, and describe the actual validation performance at higher resolution. Vertical lines outline the number of variables selected in MUVR 'min', 'mid', and 'max' models on the x-axis. **(c)** Histogram and density curve outlining the distribution of misclassifications by 500 permuted PLS models in which the Y response vector indicating sample class was randomly sampled. The number of misclassifications by the actual PLS model with the correct Y response vector is outlined on the x-axis by the blue dashed line. A p-value derived from a student's t-test comparing the number of misclassifications between actual and permuted models is presented adjacent to the histogram and density curve. MUVR, multivariate methods with unbiased variable selection in R; NPV, negative predictive value; PLS, partial least squares; PPV, positive predictive value.

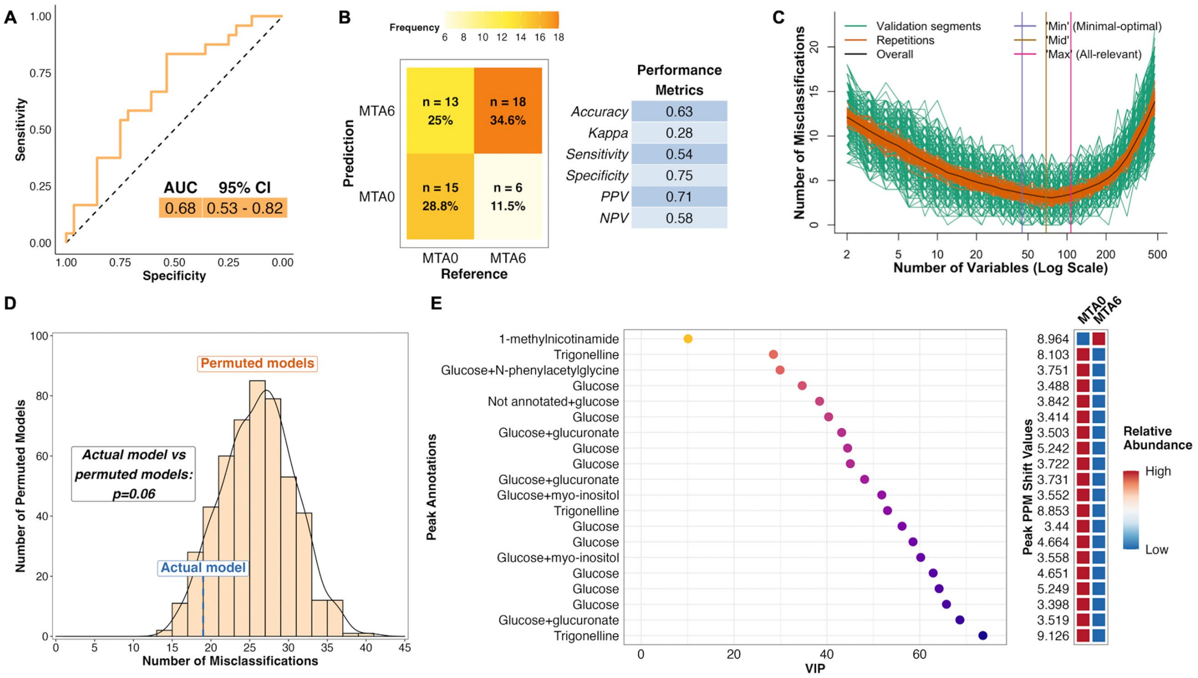

Figure S3. Multivariate analysis of changes in urinary metabolome from baseline to month 6 in the MTA arm (all available samples). Number of samples inputted to the MUVR PLS model: MTA0, n=28; MTA6, n=24. **(a)** Receiver operating characteristic curve for the MUVR PLS classification model fit to urinary ^{1}H-NMR peaks for these two groups of samples. Inset are the AUC and associated 95% CI for the curve. **(b)** Confusion matrix outlining sample classification by the PLS model. The x-axis presents the actual class of the inputted samples. The y-axis presents the sample class predicted by the models. Correctly and incorrectly classified samples are outlined on opposing diagonals of the confusion matrix. Additional performance metrics relating to sample classification at the 50% probability threshold are presented in the table adjacent to the confusion matrix. **(c)** Recursive ranking and backward elimination of variables (right-to-left on the x-axis) in the inner segments of the MUVR PLS model to achieve optimal validation performance, quantified by the number of misclassifications (y-axis). Green lines represent validation curves per inner segment and may fluctuate. Orange and black lines represent inner segment curves averaged per model repetition and overall (100 model repetitions), respectively, and describe the actual validation performance at higher resolution. Vertical lines outline the number of variables selected in MUVR 'min', 'mid', and 'max' models on the x-axis. **(d)** Histogram and density curve outlining the distribution of misclassifications by 500 permuted PLS models in which the Y response vector indicating sample class was randomly sampled. The number of misclassifications by the actual PLS model with the correct Y response vector is outlined on the x-axis by the blue dashed line. A p-value derived from a student's t-test comparing the number of misclassifications between actual and permuted models is presented adjacent to the histogram and density curve. **(e)** Dotplot of the top 20 most important annotated peaks to performance of the PLS model ranked by VIP values. Multiple peaks were identified for certain metabolites. When multiple metabolites are present in a given peak, metabolites are listed in order of relative abundance in the peak, with the most abundant metabolite listed first. The adjacent heatmap illustrates relative abundance of metabolites based on mean peak intensity across all samples in the two groups evaluated by the PLS model. 95% CI, 95% confidence interval; AUC, area under the receiver operating characteristic curve; MTA0, baseline samples from MTA group; MTA6, month 6 samples from MTA group; MUVR, multivariate methods with unbiased variable selection in R; NMR, nuclear magnetic resonance spectroscopy; NPV, negative predictive value; PLS, partial least squares; PPM, parts per million chemical shift relative to TSP-d4; PPV, positive predictive value; VIP, variable importance in projection.

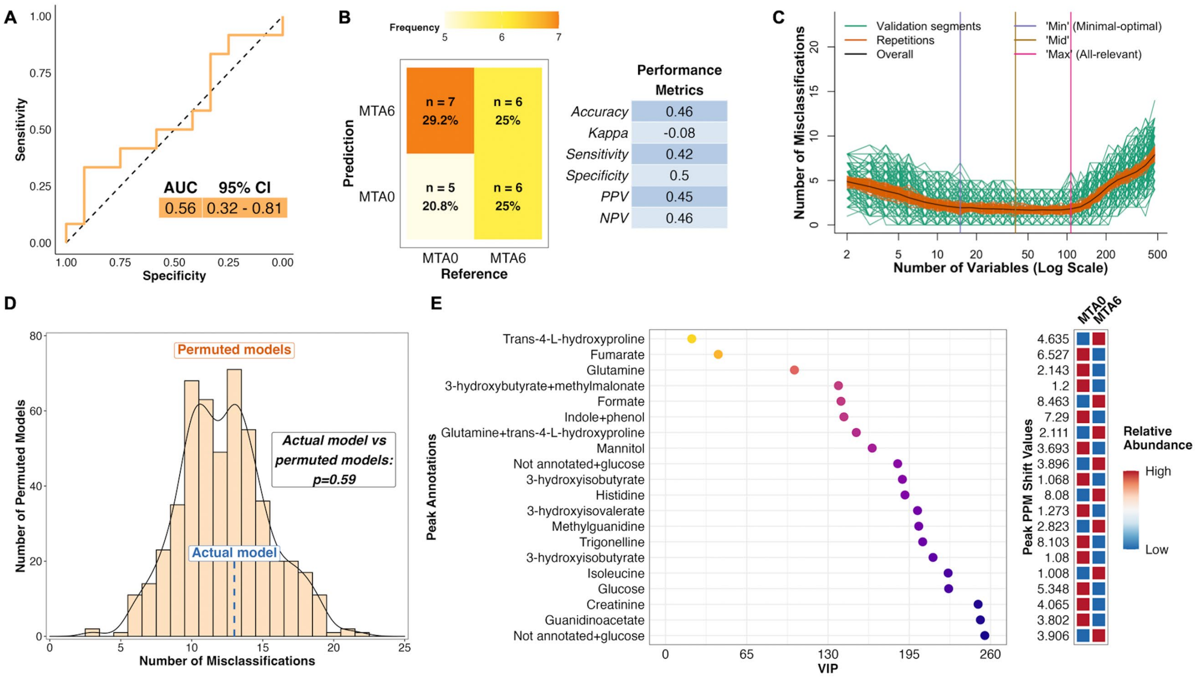

Figure S4. Multivariate analysis of changes in the urinary metabolome from baseline to month 6 in the MTA arm (paired samples only). Number of samples inputted to the MUVR PLS model: MTA0, n=12; MTA6, n=12. **(a)** Receiver operating characteristic curve for the MUVR PLS classification model fit to urinary ^{1}H-NMR peaks for these two groups of samples. Inset are the AUC and associated 95% CI for the curve. **(b)** Confusion matrix outlining sample classification by the PLS model. The x-axis presents the actual class of the inputted samples. The y-axis presents the sample class predicted by the models. Correctly and incorrectly classified samples are outlined on opposing diagonals of the confusion matrix. Additional performance metrics relating to sample classification at the 50% probability threshold are presented in the table adjacent to the confusion matrix. **(c)** Recursive ranking and backward elimination of variables (right-to-left on the x-axis) in the inner segments of the MUVR PLS model to achieve optimal validation performance, quantified by the number of misclassifications (y-axis). Green lines represent validation curves per inner segment and may fluctuate. Orange and black lines represent inner segment curves averaged per model repetition and overall (100 model repetitions), respectively, and describe the actual validation performance at higher resolution. Vertical lines outline the number of variables selected in MUVR ‘min’, ‘mid’, and ‘max’ models on the x-axis. **(d)** Histogram and density curve outlining the distribution of misclassifications by 500 permuted PLS models in which the Y response vector indicating sample class was randomly sampled. The number of misclassifications by the actual PLS model with the correct Y response vector is outlined on the x-axis by the blue dashed line. A p-value derived from a student’s t-test comparing the number of misclassifications between actual and permuted models is presented adjacent to the histogram and density curve. **(e)** Dotplot of the top 20 most important annotated peaks to performance of the PLS model ranked by VIP values. Multiple peaks were identified for certain metabolites. When multiple metabolites are present in a given peak, metabolites are listed in order of relative abundance in the peak, with the most abundant metabolite listed first. The adjacent heatmap illustrates relative abundance of metabolites based on mean peak intensity across all samples in the two groups evaluated by the PLS model. 95% CI, 95% confidence interval; AUC, area under the receiver operating characteristic curve; MTA0, baseline samples from MTA group; MTA6, month 6 samples from MTA group; MUVR, multivariate methods with unbiased variable selection in R; NMR, nuclear magnetic resonance spectroscopy; NPV, negative predictive value; PLS, partial least squares; PPM, parts per million chemical shift relative to TSP-d4; PPV, positive predictive value; VIP, variable importance in projection.

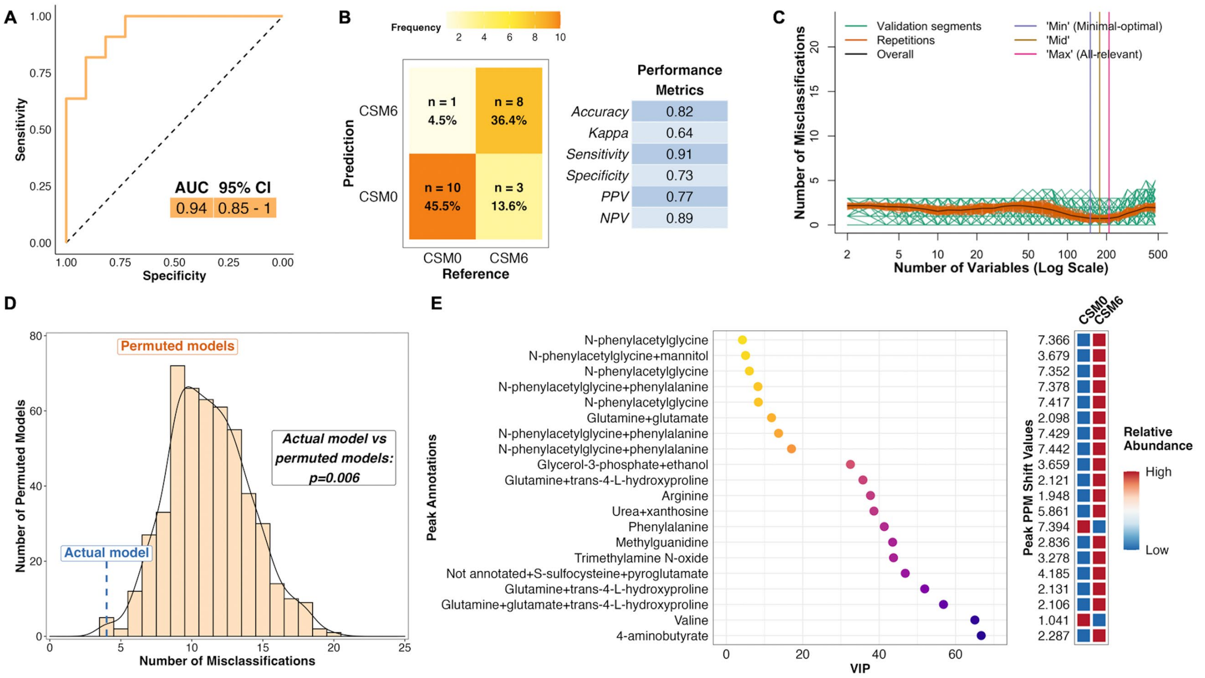

Figure S5. Multivariate analysis of changes in the urinary metabolome from baseline to month 6 in the CSM arm (paired samples only). Number of samples inputted to the MUVR PLS model: CSM0, n=11; CSM6, n=11. **(a)** Receiver operating characteristic curve for the MUVR PLS classification model fit to urinary ^{1}H-NMR peaks for these two groups of samples. Inset are the AUC and associated 95% CI for the curve. **(b)** Confusion matrix outlining sample classification by the PLS model. The x-axis presents the actual class of the inputted samples. The y-axis presents the sample class predicted by the models. Correctly and incorrectly classified samples are outlined on opposing diagonals of the confusion matrix. Additional performance metrics relating to sample classification at the 50% probability threshold are presented in the table adjacent to the confusion matrix. **(c)** Recursive ranking and backward elimination of variables (right-to-left on the x-axis) in the inner segments of the MUVR PLS model to achieve optimal validation performance, quantified by the number of misclassifications (y-axis). Green lines represent validation curves per inner segment and may fluctuate. Orange and black lines represent inner segment curves averaged per model repetition and overall (100 model repetitions), respectively, and describe the actual validation performance at higher resolution. Vertical lines outline the number of variables selected in MUVR 'min', 'mid', and 'max' models on the x-axis. **(d)** Histogram and density curve outlining the distribution of misclassifications by 500 permuted PLS models in which the Y response vector indicating sample class was randomly sampled. The number of misclassifications by the actual PLS model with the correct Y response vector is outlined on the x-axis by the blue dashed line. A p-value derived from a student's t-test comparing the number of misclassifications between actual and permuted models is presented adjacent to the histogram and density curve. **(e)** Dotplot of the top 20 most important annotated peaks to performance of the PLS model ranked by VIP values. Multiple peaks were identified for certain metabolites. When multiple metabolites are present in a given peak, metabolites are listed in order of relative abundance in the peak, with the most abundant metabolite listed first. The adjacent heatmap illustrates relative abundance of metabolites based on mean peak intensity across all samples in the two groups evaluated by the PLS model. 95% CI, 95% confidence interval; AUC, area under the receiver operating characteristic curve; CSM0, baseline samples from CSM group; CSM6, month 6 samples from CSM group; MUVR, multivariate methods with unbiased variable selection in R; NMR, nuclear magnetic resonance spectroscopy; NPV, negative predictive value; PLS, partial least squares; PPM, parts per million chemical shift relative to TSP-d4; PPV, positive predictive value; VIP, variable importance in projection.

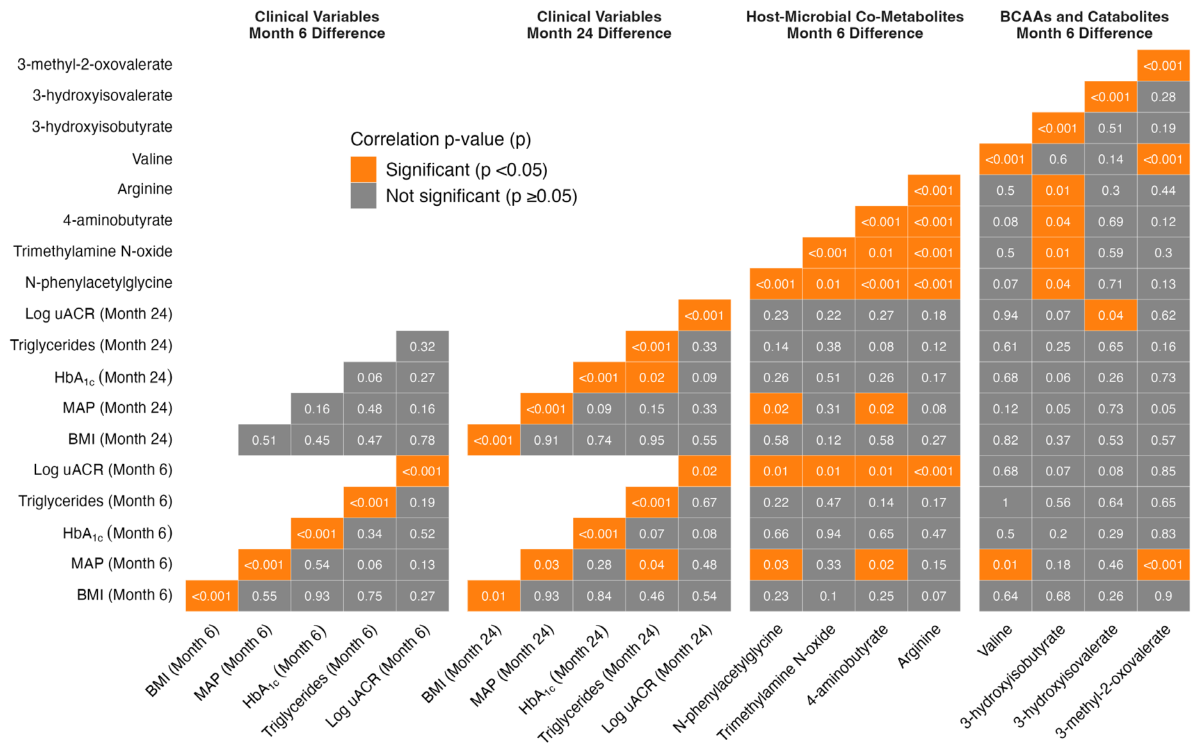

Figure S6. Matrix of p-values for Pearson correlations between changes in clinical parameters at months 6 and 24 with changes in urinary metabolites reflective of host-microbial co-metabolism and BCAA catabolism at month 6. The p-values displayed correspond to Pearson correlation r values outlined in **Figure 7**. Matrix cells are coloured by p-value categories as follows: significant ($p < 0.05$), orange; not significant ($p \geq 0.05$), grey. Absolute differences in clinical parameters at both timepoints (months 6 and 24) were correlated with absolute differences in PQN-normalised metabolite abundance at month 6. Correlation p-values were computed using the function 'cor_pmat' from the R package ggcorrplot. Correlations were performed for the subset of study participants in the CSM arm with paired urinary ^{1}H-NMR data available at baseline and month 6 (n=11). BCAA, branched-chain amino acid; BMI, body-mass index; CSM, combined metabolic surgery plus medical therapy; HbA_{1c}, glycated haemoglobin; MAP, mean arterial pressure; PQN, probabilistic quotient normalization; uACR, urinary albumin-to-creatinine ratio.

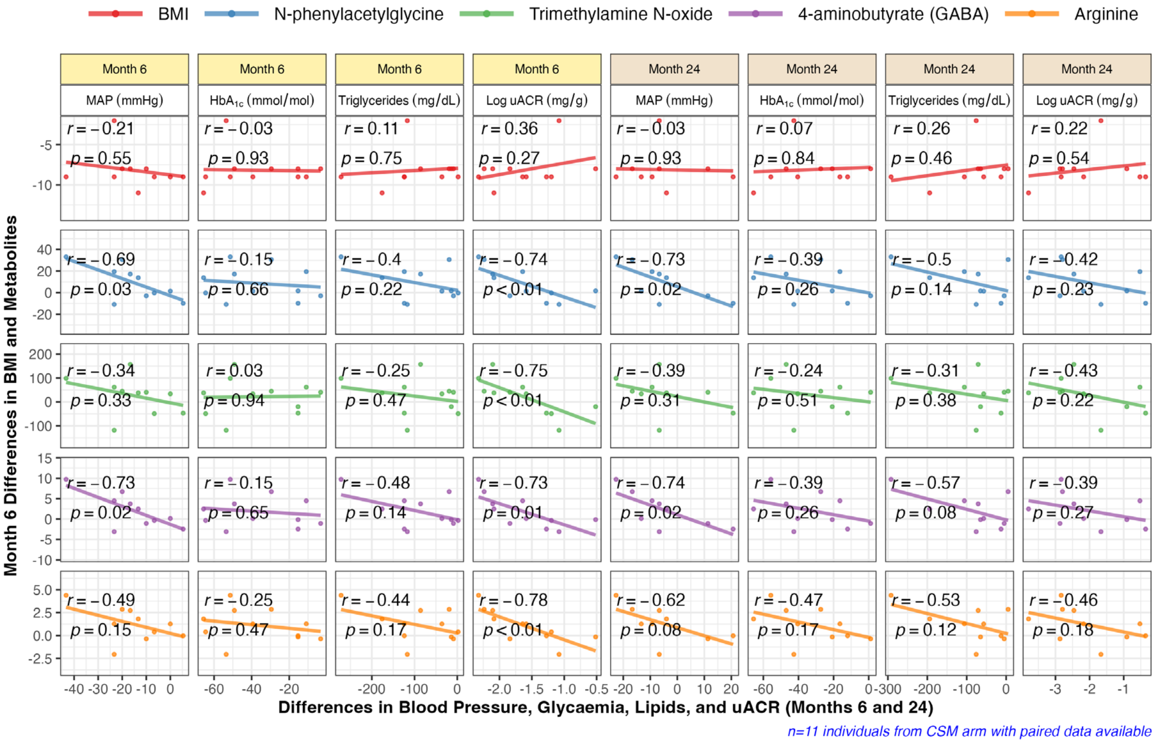

Figure S7. Scatterplots of month 6 changes in BMI and in urinary host-microbial co-metabolites with changes in blood pressure, glycaemia, lipids, and uACR in the CSM arm during follow-up. Absolute differences in blood pressure, glycaemia, lipids, and uACR from baseline to follow-up (month 6 and month 24) are presented on the x-axis. Absolute differences in BMI and in urinary metabolites from baseline to month 6 are presented on the y-axis. Plots are facetted by clinical parameters (MAP, HbA_{1c}, triglycerides, and log uACR) as well as follow-up time (month 6 and month 24). Correlations were performed for the subset of study participants in the CSM arm with paired urinary ^{1}H-NMR data available at baseline and month 6 (n=11). Linear model regression lines of best fit for the data points are presented. Pearson r correlation values between changes in BMI/metabolites and changes in clinical parameters as well as associated p-values are displayed on the plots. BMI, body-mass index; CSM, combined metabolic surgery plus medical therapy; HbA_{1c}, glycated haemoglobin; MAP, mean arterial pressure; uACR, urinary albumin-to-creatinine ratio.

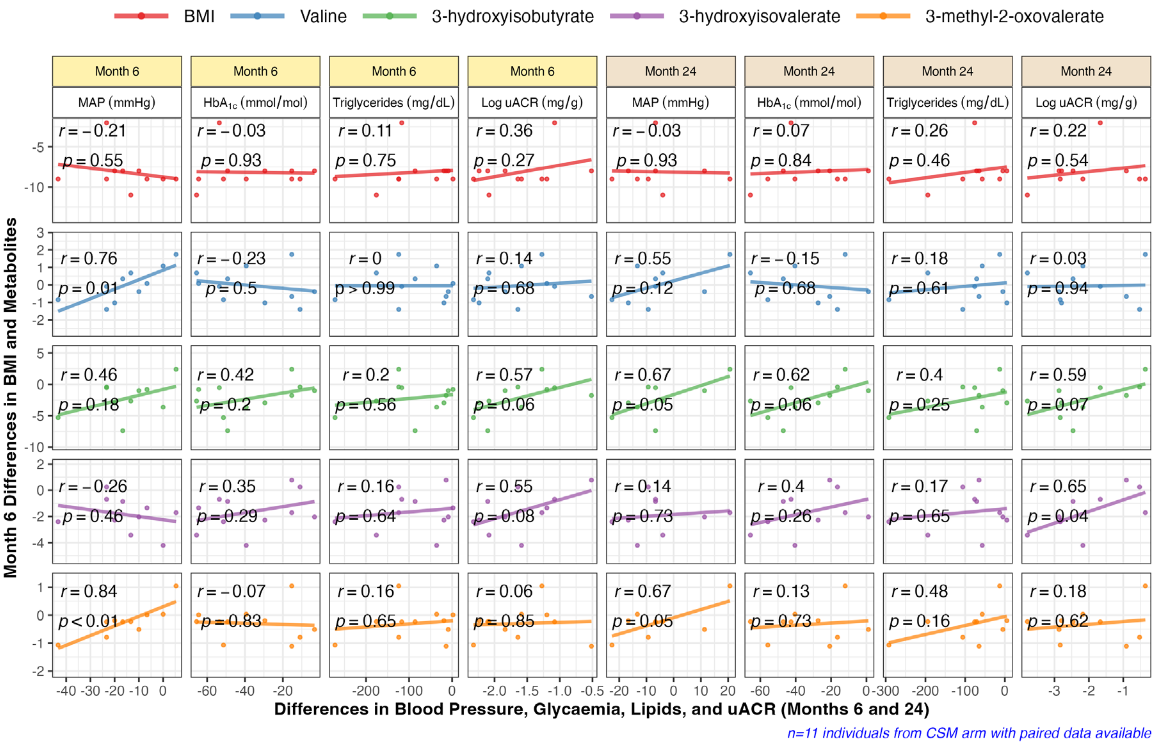

Figure S8. Scatterplots of month 6 changes in BMI and in urinary metabolites reflective of BCAA catabolism with changes in blood pressure, glycaemia, lipids, and uACR in the CSM arm during follow-up. Absolute differences in blood pressure, glycaemia, lipids, and uACR from baseline to follow-up (month 6 and month 24) are presented on the x-axis. Absolute differences in BMI and in urinary metabolites from baseline to month 6 are presented on the y-axis. Plots are facetted by clinical parameters (MAP, HbA_{1c}, triglycerides, and log uACR) as well as follow-up time (month 6 and month 24). Correlations were performed for the subset of study participants in the CSM arm with paired urinary ^{1}H-NMR data available at baseline and month 6 (n=11). Linear model regression lines of best fit for the data points are presented. Pearson r correlation values between changes in BMI/metabolites and changes in clinical parameters as well as associated p-values are displayed on the plots. BMI, body-mass index; CSM, combined metabolic surgery plus medical therapy; HbA_{1c}, glycated haemoglobin; MAP, mean arterial pressure; uACR, urinary albumin-to-creatinine ratio.
